# Research on the method of travel area clustering of urban public transport based on Sage-Husa adaptive filter and improved DBSCAN algorithm

**DOI:** 10.1371/journal.pone.0259472

**Published:** 2021-12-22

**Authors:** Xinhuan Zhang, Les Lauber, Hongjie Liu, Junqing Shi, Jinhong Wu, Yuran Pan

**Affiliations:** 1 College of Engineering, Zhejiang Normal University, Jinhua, Zhejiang Province, China; 2 Kansas Public Employee Retirement System, Topeka, Kansas, United States of America; 3 School of Electronic and Information Engineering, Xi’an Jiao Tong University, Xi’an, Shanxi Province, China; Valahia University of Targoviste: Universitatea Valahia din Targoviste, ROMANIA

## Abstract

The travel trajectory data of mobile intelligent terminal users are characterized by clutter, incompleteness, noise, fuzzy randomness. The accuracy of original data is an essential prerequisite for better results of trajectory data mining. The Density-Based Spatial Clustering of Applications with Noise (DBSCAN) is one of the most effective trajectory data mining methods, but the selection of input parameters often limits it. The Sage-Husa adaptive filtering algorithm effectively controls the error range of mobile phone GPS data, which can meet the positioning accuracy requirements for DBSCAN spatial clustering having the advantages of low cost and convenient use. Then, a novel cluster validity index was proposed based on the internal and external duty cycle to balance the influence of the distance within-cluster, the distance between clusters, and the number of coordinate points in the process of clustering. The index can automatically choose input parameters of density clustering, and the effective clustering can be formed on different data sets. The optimized clustering method can be applied to the in-depth analysis and mining of traveler behavior trajectories. Experiments show that the Sage -Husa adaptive filtering algorithm proposed further improves the positioning accuracy of GPS, which is 17.34% and 15.24% higher eastward and northward, 14.25%, and 18.17% higher in 2D and 3D dimensions, respectively. The number of noise points is significantly reduced. At the same time, compared with the traditional validity index, the evaluation index based on the duty cycle proposed can optimize the input parameters and obtain better clustering results of traveler location information.

## I. Introduction

The site selection and layout of the stops are an essential part of urban public transport service design. To attract more travelers to choose public transport, managers need to consider passenger travel needs, formulate reasonable stops and routes layout so that walking distance to the stops should not be too large, to maximize the advantages of the public transport. Usually, the travel needs with similar origins or destinations are grouped into one category through spatial clustering, and the appropriate location should be selected in the public transport travel area after clustering. Then, the walking distance between users and stops in the region is within a reasonable range.

The popularity of intelligent mobile terminals makes it possible to obtain massive and high-precision Spatio-temporal data. Meanwhile, the development of mobile internet accelerates the generation and accumulation of Spatio-temporal data. Hundreds of millions of mobile location data are generated daily from different sources, such as smartphones equipped with GPS navigation devices, providing a database for research into users’ travel trajectory patterns. The travel trajectory data of mobile intelligent terminal users are characterized by clutter, incompleteness, noise, and fuzzy randomness. The accuracy of the original data is an essential premise for achieving a better result. Therefore, exploring a more accurate and efficient anti-noise GPS data preprocessing method suitable for mobile smartphone users’ trajectory based on the existing algorithm is the basis of current research on travel habits, dwell characteristics, and related application development, which is an essential prerequisite for travel trajectory data mining.

In addition, based on the trajectory data of urban public transport APP, the traditional DBSCAN (Dense-Based Spatial Clustering of Applications with Noise) Clustering algorithm was improved in the paper: a novel cluster validity index was proposed based on internal and external duty cycle to balance the influence of the distance within-cluster, the distance between clusters, and the number of coordinate points in the process of clustering. The index can automatically choose input parameters of density clustering, and the effective clustering can be formed on different data sets. The optimized clustering method can be applied to the in-depth analysis and mining of traveler behavior trajectories.

This study aims to explore the extraction scheme about spatial dwell point suitable for mobile intelligent terminal users’ trajectory. A more accurate, efficient, and noise-resistant mobile phone GPS data preprocessing method makes it possible to efficiently mine travel trajectory data; The improved DBSCAN algorithm can optimize the input parameters and obtain better clustering results of traveler location information. The combination of the two can significantly improve the reliability of the identification results of bus travel areas. According to the results of travel area identification, the site settings are optimized to reduce the walking distance of travelers, improve the travel experience and service reliability, save travel costs, and provide data support for the construction of urban public transport networks of wisdom cities.

## II. Literature review

### A. Sage-Husa adaptive filtering

Sage and Husa jointly proposed Sage-Husa adaptive filtering algorithm. It is a variance matching algorithm proposed based on the Kalman filtering algorithm. This method can estimate the first and second moments of the current noise more accurately, and the algorithm has a simple principle and high real-time performance. This filtering algorithm has been widely used in many fields. In the process of recursion and filtering, to achieve the optimal filtering performance (filtering accuracy, stability, real-time), Sage-Husa adaptive filtering algorithm uses the observed value to modify the predicted value not only constantly but also constantly adjusts the system model parameters and the statistical parameters of the noise model.

Chunmei Huang et al. [1] proposes an initial alignment method based on the Sage-Husa adaptive filter, which uses observed data, online estimation noise statistical characteristics, and state simultaneously to improve the filter continuously. Hence, the filter has a higher estimation accuracy than the conventional Kalman filter. By simulating verifying, the adaptive Kalman filter enhances the convergence speed and alignment accuracy effectively. Feng Sun et al. [2] proposes a new type of Sage-Husa-based adaptive filtering algorithm on the initial alignment method of inertial, which uses observed data and automatic for online estimation and correction of statistical characteristics of noise. Simulation results show that the algorithm for improving alignment accuracy. Chihang Zhao et al. [3] proposes using the Sage-Husa adaptive Kalman filter to denoise the JSD-I/A quartz flexural gravitational sensor signal. M. Narasimhappa et al. [4] use a modified Sage-Husa adaptive Kalman filter to denoise the Fiber Optic Gyroscope (FOG) signal. The performance of the proposed algorithm is compared with the conventional Kalman filter, and the simulation results show that the modified Sage-Husa adaptive Kalman filter (SHAKF) algorithm outperforms the traditional Kalman filter technique while denoising FOG. P. Yuzhen et al. [5] proposes an adaptive extended Kalman filter (AEKF) algorithm to resolve error accumulation in mobile robot localization. They use the Sage-Husa time-varying noise estimator to estimate observation noise in real-time. Convergence and complexity of operation of AEKF are analyzed, and the experiments show that AEKF has an excellent comprehensive performance in terms of speed and precision. Q. He et al. [6] propose an improved adaptive filtering algorithm. It uses the filtering convergence criterion based on simplifying the Sage-Husa filter. Through fusing the data from a multi-sensor, more accurate information may be used in the algorithm. M. Narasimhappa et al. [7] modify the Sage-Husa Adaptive Robust Kalman Filter (MSHARKF) based on robust estimation and a time-varying statistical noise estimator. An adaptive scale factor (a) is developed based on a three-segment approach in the proposed algorithm. In the MSHARKF, the adaptive factor is updated in each iteration step. The MSHARKF algorithm is applied to minimize the bias drift and random noise of the MEMS IMUs signals. From the Allan variance analysis, the noise coefficients such as bias instability and drift are evaluated before and after minimizing. Simulation results reveal that the proposed algorithm performs better than other algorithms for similar tasks. M. Yuan et al. [8] propose an algorithm based on the Sage-Husa Kalman filter is given for navigation. The algorithm uses a polarized light camera and a magnetometer to obtain the heading angles information, a satellite receiver to collect speed and position information, and use these messages to estimate and adjust the filter parameters. Two land vehicle tests were conducted, and the heading angles were increased by 20.49% and 9.59%, respectively, which proved the effectiveness and availability of the given method. H. Yin et al. [9] proposed an improved adaptive unscented Kalman filter algorithm (AUKF), combined with lidar positioning data and the result of track estimation, which can avoid the optimal solution that the laser positioning result may trap and reduce the influence of errors caused by wheel slip and other factors. At the same time, different weights are set for the data obtained by various sensors. Combined with the Sage-Husa adaptive thought, the measurement noise characteristics can be updated. The simulation results show that, under the condition that the statistical features of the measurement noise are unknown, AUKF has higher accuracy than the traditional UKF algorithm in positioning results and fits the actual trajectory.

From the above analysis, Sage-Husa adaptive filtering performs real-time estimation of the current observation noise through the recursive method. In the estimation process, updated weights are used to modify the observation noise variance in real-time. The model error can be reduced, the divergence of the filter can be suppressed, and the filter’s accuracy can be improved by the accurate real-time estimation of the variance of observation noise. After using this algorithm, the number of noise points is reduced obviously. It has the advantages of low cost and convenient use and can meet positioning accuracy requirements for DBSCAN spatial clustering.

However, in most of the above studies, single positioning data is used as the processing data set. However, in the case of GPS big data of mobile smartphones, the data denoising results will be different to some extent. In this paper, mobile smartphone positioning has certain advantages in initial data collection methods and collection environment, and smartphone positioning may be more suitable for studying mobile intelligent terminal users’ trajectories. The denoising algorithm mentioned in this paper is applied to the GPS of the mobile smartphone to improve the precision of GPS positioning with low cost and good robustness.

### B. DBSCAN trajectory clustering

Trajectory clustering is a kind of trajectory pattern mining. Trajectory clustering aims to find the representative path or common trend of different moving objects [10]. Many different types of literature have adopted other methods to achieve the goal of trajectory mining. Cheng et al. [11] divided the trajectory into sub-trajectory segments and then applied a density-based clustering algorithm to cluster the sub-trajectory and dig out hot spots. Wang [12] proposed a grid-based motion trajectory mining algorithm. Firstly, data were divided based on the grid, and then DBSCAN was used to cluster each grid. Since the number of clusters is the required input for the fuzzy means clustering (FCM), Choong et al. [13] specify three numbers as parameters. However, the above methods only slice or mesh the trajectory data and then apply the clustering algorithm to the actual trajectory clustering scene. Because the clustering algorithm has not been improved, the clustering parameters are not accurate enough to achieve optimal results.

DBSCAN is widely used in many scientific fields due to its simplicity and ability to detect clusters of different sizes and shapes. Because the traditional DBSCAN algorithm relies heavily on the user’s manual experience when selecting the clustering parameters. If the user does not have enough knowledge to determine the parameter values correctly, the improper value of input parameters may affect the quality of the clustering results. To overcome this defect, on the one hand, some researchers combined the two methods to determine parameters, Sharma et al. [14] combined with K-nearest neighbor with DBSCAN to realize parameter-free clustering technology. Hou et al. [15] mixed DSets (dominant sets) with DBSCAN to search values automatically, but these methods need to process data at least twice. Complex steps are not suitable for large-scale data.

On the other hand, improving the validity index of the clustering algorithm can also effectively select the clustering parameters and enhance the clustering effect. Duun Validity Index (1973) [16], DBI (Davis-Bouldin index) (1979) [17], and Silhouette Coefficient (1999) [18] are the three fundamental indexes to evaluate the unlabeled clustering algorithm. Zhou et al. [19] designed a new clustering validity index, called compact-separate proportion (CSP) index, to assess the clustering results generated by the AHC algorithm and determine the optimal number of clustering. Karo et al. [20] proposed a spatial regional clustering validity index that modified Davies Bouldin Index using Polygon Dissimilarity Function (PDF). Acharya et al. [21] introduced a new distance validity index based on line symmetry to define four known clustering validity indicators. Thomas et al. [22] proposed that cylindrical distance is used instead of Euclidean space, which tries to capture the data density along the connecting mean line segment to estimate the distance between the cluster means. However, the existing validity index is generally targeted at 2D synthetic data sets. It only focuses on the degree of clustering cohesion and clustering spacing while ignoring the clustering density, which means that the clustering results of these methods may become a long bar clustering, which is not reasonable in real life. Because of the defects of these indexes, it is necessary to improve DBSCAN and its validity index at the same time to find out the optimal cluster number of traveler location information data set.

## III. Problem description

### A. Travel trajectory problems of urban residents

The traditional research method of urban residents’ travel trajectory is done by questionnaire survey and offline visit, which requires a lot of human and material resources and is susceptible to the subjective concept of investigators, resulting in deviation of trajectory analysis. The development of the internet and big data technology makes it possible to analyze the travel trajectory of a single individual from massive amounts of data. Familiar information sources include signaling data of the mobile phone, IC data, APP data of the mobile phone. The Global Positioning System (GPS) information points of travelers can be obtained through data collection, and the transportation distribution of a single individual can be obtained by superimposing the GPS information points of a single individual.

Travel has two contradictory characteristics of regularity and randomness. In most cases, most people’s travel has a frequency, usually manifested by the periodic characteristics of travel for the workday and random features of the trip for non-workday. Other groups, such as children, the elderly, and freelancers, show spontaneous travel throughout the day.

The travel distribution data of residents are represented by numerous discrete information points, each representing the spatial location of residents at a specific moment. Due to the regularity and randomness of residents’ travel, different travelers will eventually form travel point groups with different densities and sizes. According to other clustering methods, the point groups formed are also different. The cluster results determine whether the user travel analysis is accurate to a certain extent, and different clustering methods can get different clustering results. How to efficiently and accurately cluster the travel point group is one of the problems we are faced with.

### B. Clustering method of urban residents’ travel trajectories

There are six commonly used clustering methods, and their advantages and disadvantages are listed as follows:

1. **Partition-based methods:** it is an older clustering method, which is widely used at present. It is simple and efficient for large data sets, with low time and space complexity. However, it is easy to form an optimal solution, which is too sensitive to noise and outliers, so it is necessary to set the ***K*** value in advance. Different ***K*** values may cause different clustering results. Secondly, this method may hide non-convex or clusters of very different sizes.
2. **Hierarchical methods:** are highly interpretable and easy to understand and implement, but the time complexity is too high, usually O (n^3^).
3. **Grid-based methods:** The clustering speed is very fast because the clustering speed is independent of the number of data objects and depends on the number of units in each dimension of the data space. Its disadvantages are being sensitive to parameters, being unable to deal with irregular distribution of data. The efficiency of this clustering algorithm is at the cost of the accuracy of clustering results.
4. **Model-based methods:** The classification of clustering is expressed in probabilistic modes, and parameters can describe the features of each cluster with high adaptability. Its disadvantage is that the execution efficiency is not high, especially when the number of distributions is large and the data is small.
5. **Fuzzy clustering method:** it has a good clustering effect for data satisfying normal distribution, but it cannot ensure that FCM converges to an optimal solution, and the performance of the algorithm depends on the initial clustering center.
6. **Density-based methods:** They are not sensitive to noise and can find clusters of arbitrary shapes. However, the clustering results are closely related to the parameters. In the face of the different sparse degrees of clustering, the same judgment criteria will destroy the natural structure of clustering.

Combined with the characteristics of residents’ travel data and compared with the features and applicability of the above six standard clustering algorithms, the density clustering algorithm is more suitable for studying urban residents’ travel original-destination clustering. The most common algorithm in density clustering is the DBSCAN algorithm, which can be adapted to data clustering of any shape, effectively remove noise interference, and generate a specified number of clustering results according to the features of data points. However, at the same time, DBSCAN has the common disadvantages of the density clustering algorithm. The clustering results are directly related to the parameter selection., selecting the input parameters carefully to obtain the optimal outcome of travel trajectory clustering and how to choose the input parameters scientifically and effectively is one of the problems that should be solved in this paper.

## IV. Methodology

Firstly, Sage-Husa adaptive filtering is used to reduce the error range of GPS data and improve the accuracy of mobile GPS positioning at a low cost. Then, the automatic selection of DBSCAN input parameters is realized according to the enhanced DBSCAN algorithm and the novel validity index of trajectory clustering results. This index balances the degree of cohesion, the distance between clusters, and the density within groups. It calculates the optimal input parameter of the density clustering model to avoid the limitation of manual parameters. After comparing the 2D synthetic data with the case travel data of public transport of Jinhua city, the evaluation index is superior to the traditional evaluation index in the density-based geographical location information clustering.

### A. Basic theory

#### 1) Sage-Husa adaptive filtering

*i*. *Basic principle*. The Sage-Husa adaptive filtering performs real-time estimation of the current observation noise by the recursive method. In the estimation process, the updated weight is used to modify the observation noise variance in real-time. The model error can be reduced, the divergence of the filter can be suppressed, and the filter’s accuracy can be improved by the real-time accurate estimation of the variance of observation noise. First, consider the following random linear offline system (see Eq (1)):

$$\{\begin{array}{c} X_{t}=A_{t,t-1}\cdot X_{t-1}+C_{t,t-1}\cdot u_{t-1}+B_{t,t-1}\cdot w_{t-1}\\ Y_{t}=H_{t}\cdot X_{t}+v_{t} \end{array}$$ (Eq 1)

In Eq (1), *X*_*t*_ is the system state vector at time *t*; *A*_*t*,*t*−1_ is the state transition matrix from time *t-*1 to time *t*; *C*_*t*,*t*−1_ is the control-input matrix from time *t-*1 to time *t*, *A*_*t*,*t*−1_ and *C*_*t*,*t*−1_ can both change as time *t* changes; *Y*_*t*_ is the observation vector at time *t*, *H*_*t*_ is the observation matrix at time *t*; *w*_*t*−1_ is the process noise at time *t-*1, and *v*_*t*_ is the observation noise at time *t*, which are independent of each other, and both are normal white noise sequences with time-varying mean and covariance matrix. Among them, process noise and observation noise conform to the following statistical characteristics (see Eq (2)):

$$\{\begin{array}{c} E[w_{t}]=q_{t}\\ E[(w_{t}-q_{t}){\left(w_{j}-q_{j}\right)}^{T}]=Q_{t}\delta_{tj}\\ E[v_{t}]=r_{t}\\ E[(v_{t}-r_{t}){\left(v_{j}-r_{j}\right)}^{T}]=R_{t}\delta_{tj}\\ E[(w_{t}-q_{t}){\left(v_{j}-r_{j}\right)}^{T}]=0 \end{array}$$ (Eq 2)

Where the Sage-Husa adaptive algorithm can be described as Eq (3):

$$\{\begin{array}{c} {\hat{X}}_{t}={\hat{X}}_{t,t-1}+K_{t}∙\varepsilon_{t}\\ {\hat{X}}_{t,t-1}=A_{t,t-1}∙{\hat{X}}_{t-1}+C_{t,t-1}∙u_{t-1}+B_{t,t-1}∙{\hat{q}}_{t-1}\\ K_{t}=P_{t,t-1}∙{H_{t}}^{T}{\left[H_{t}∙P_{t,t-1}∙{H_{t}}^{T}+{\hat{R}}_{t}\right]}^{-1}\\ \varepsilon_{t}=Y_{t}-H_{t}∙{\hat{X}}_{t,t-1}-{\hat{r}}_{t}\\ P_{t,t-1}=A_{t,t-1}∙P_{t-1}∙{A_{t,t-1}}^{T}+B_{t,t-1}∙{\hat{Q}}_{t-1}∙{B_{t,t-1}}^{T}\\ P_{t,t}=[I-K_{t}∙H_{t}]∙P_{t,t-1} \end{array}$$ (Eq 3)

(Where ${\hat{q}}_{t},\;{\hat{Q}}_{t},\;{\hat{r}}_{t},\;{\hat{R}}_{t}$ can be obtained by recursion of time-varying noise estimator (see Eq (4)):

$$\{\begin{array}{c} {\hat{q}}_{t}=(1-d_{t-1}){\hat{q}}_{t-1}+d_{t-1}({\hat{X}}_{t}-A_{t,t-1}∙{\hat{X}}_{t-1})\\ {\hat{Q}}_{t}=(1-d_{t-1}){\hat{Q}}_{t-1}+d_{t-1}(K_{t}\varepsilon_{t}{\varepsilon_{t}}^{T}{K_{t}}^{T}+P_{t}-A_{t,t-1}∙P_{t-1}∙{A_{t,t-1}}^{T})\\ {\hat{r}}_{t}=(1-d_{t-1}){\hat{r}}_{t-1}+d_{t-1}(Y_{t}-H_{t}{\hat{X}}_{t,t-1})\\ {\hat{R}}_{t}=(1-d_{t-1}){\hat{R}}_{t-1}+d_{t-1}(\varepsilon_{t}{\varepsilon_{t}}^{T}-H_{t}∙P_{t,t-1}∙{H_{t}}^{T})\\ d_{t}=(1-b)/(1-b^{k+1}),\;0<b<1\;and\;b\;is\;the\;forgetting\;factor. \end{array}$$ (Eq 4)

Where, *d*_*t*_ is the update rate and represents the update rate of noise parameters.

It can be seen from the above equations that the Sage-Husa adaptive algorithm estimates and corrects the statistical characteristics of process noise and observation noise in real-time while using GPS measurement data for recursive filtering to achieve the purpose of adaptive filtering. The method is simple in principle and good in real-time, so it has been widely applied in many fields. According to the above algorithm, it is easy to implement the computer program, and the autoregressive operation can be continued.

*ii*. *Basic steps*. When using Sage-Husa adaptive filtering to de-noise GPS positioning data, the basic steps are as follows:

1. Firstly, the state equation and the observation equation are established, and then the observation vector is determined.
2. The prior data were used to calibrate and test the model, and then the model noise covariance *w*_*t*−1_ and *v*_*t*_ was determined.
3. The model was validated with measured data.

**Fig 1:**
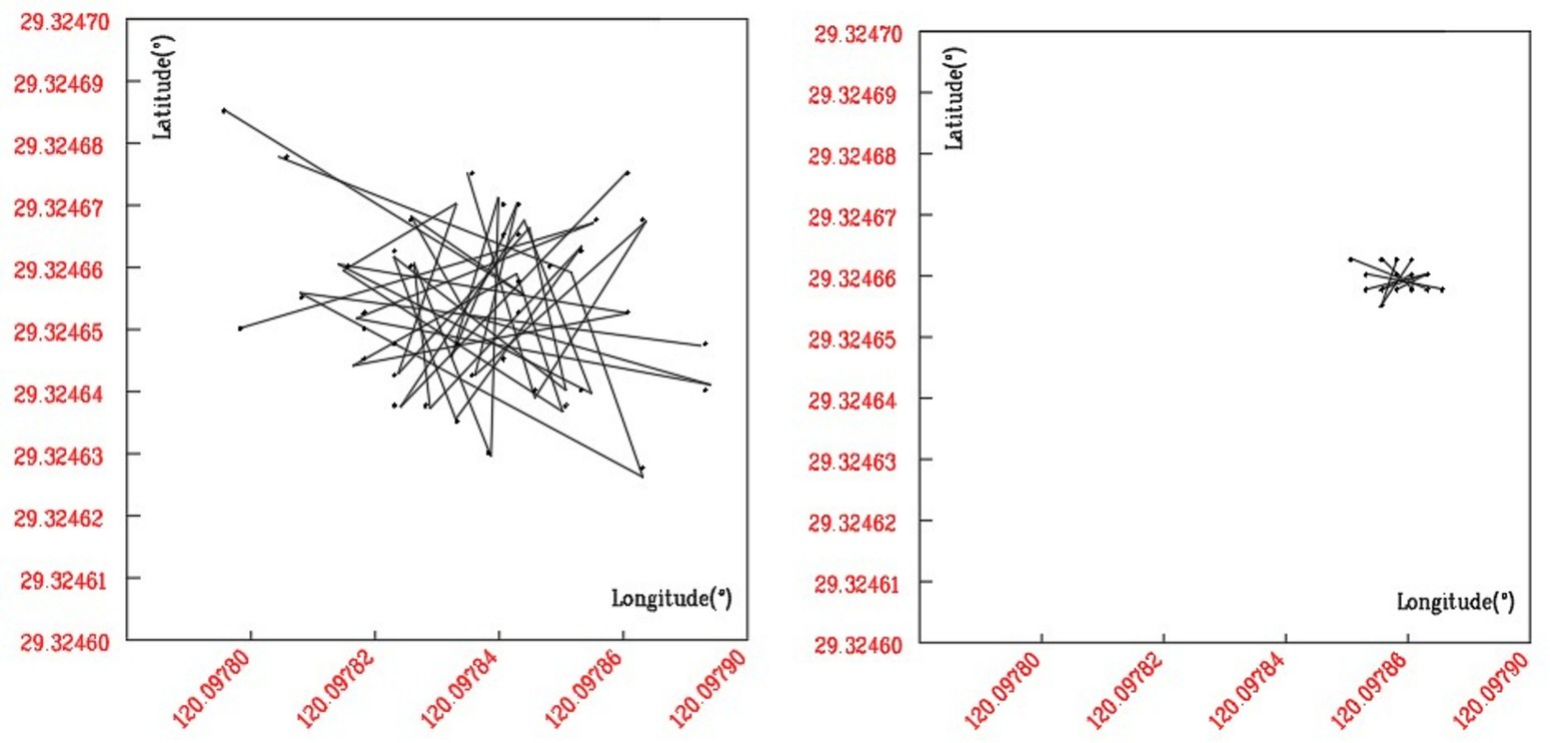
GPS data distribution before and after SAGE-HUSA adaptive filtering.

We used Sage-Husa adaptive filtering algorithm to process and analyze the data collected by the mobile phone GPS (as shown in Fig 1(a) is the distribution map of original GPS data; Fig 1(b) is the distribution map of GPS data after Sage-Husa adaptive filtering. According to the experimental analysis, the significant data fluctuation can be effectively eliminated by Sage-Husa adaptive filtering, and the mean error of longitude and latitude is controlled at 5.2m.

#### 2) DBSCAN algorithm

Density-based Spatial Clustering of Applications with Noise (DBSCAN) algorithm is a representative density-based Clustering analysis algorithm, which can find arbitrary shapes of clusters and effectively shield interference from noise data.

*i*. *Working principle*. DBSCAN algorithm uses the high-density connectivity of clusters to find clusters of arbitrary shape quickly. Its core idea is that for each object constituting the cluster, the number of objects in its *Eps* neighborhood must not be less than a given value *MinPts*. The following are some definitions involved in the basic idea of density-based clustering:

1. ***Eps*:** the distance set by the user is *Eps*. the object is in the area with itself as the dot and *Eps* as the radius called the *Eps* neighborhood of the object.
2. ***Core object*:** an object is a core object if the number of data points in its *Eps* neighborhood is greater than or equal to the given value *MinPts*.
3. ***Direct density reachable*:** in sample set *D*, *p* is the core object, *q* is in the *Eps* neighborhood of *p*, and object *q* is directly density reachable from object *p*.
4. ***Density Reachable*:** in sample set *D*, a series of sample points *P*_1_, *P*_2_, *P*_*n*_,…, *P*_*1*_ = *p*, *P*_*n*_ = *q*, *P*_*i*_∈*D*, 1≤ *i* ≤ *n*, if object *P*_*i*_ is direct density reachable from the *P*_*i*-1_, then object *p* is density reachable from object *q*, this relationship is asymmetric.
5. ***Density connected*:** If there is an object ***O*** in object set ***D***, objects ***p*** and ***q*** are density-connected if object ***O*** is density-reachable to objects ***p*** and ***q***. This relationship is symmetric.
6. ***Noise*:** a density-based cluster is a collection of the largest density-connected objects based on density-reachability. Objects that are not contained in any cluster are called noise.

In the practical application of urban site location selection, *Eps* can represent the distance from the residence or workplace to the stops of users who choose to take urban public transport. *MinPts* can define the minimum number of covered passengers required to meet the stop setting conditions. Density-connected points represent all users served by any stop. The density reachability relationship is the basis for the DBSCAN algorithm to divide the travel demand of the same passengers. The density connected point represents the passenger travel cluster that can be divided into one cluster.

*ii*. *Steps in the DBSCAN algorithm*. Based on the above definition, the DBSCAN algorithm uses a method to find density reachable objects for clustering analysis. The correctness of the DBSCAN algorithm is guaranteed because a cluster is equivalent to the set ***O*** consisting of all the density connected points objects of any core object in the cluster.

DBSCAN finds all density-reachable objects contained in each cluster by iteratively looking for all directly density-reachable objects. The specific methods are as follows:

**Input: database *D*, *Eps*, *Minpts* (given minimum number);**

**Output: cluster**.

1. Check the unchecked object ***p*** in the database, and if ***p*** has not been processed (grouped into a cluster or marked as noise), check its *Eps* neighborhood *N*_*Eps*_(***p***), if the number of objects contained is not less than *MinPts*, a new cluster ***C*** is created, and add all points of *N*_*Eps*_*(****p****)* to *C*;
2. For all unchecked objects ***q*** in ***C***, check its *Eps* neighborhood *N*_*Eps*_(***q***), if *N*_*Eps*_*(q)* contains at least *MinPts* objects, then objects of *N*_*Eps*_*(q)* that do not belong to any cluster are added to ***C***;
3. Repeat step (2) to continue checking for unchecked objects in ***C*** until no new objects are added to the current cluster ***C***;
4. Repeat steps (1) through (3) until all objects fall into a cluster or are marked as noise.

The density reachable objects are obtained by continuously executing region queries to achieve a region query and return all objects in the specified region.

*iii*. *Disadvantages of the DBSCAN algorithm*. DBSCAN needs to manually set two parameters, *Eps* and *Minpts*, which are set by the user and remain the same during the run. When *Eps* is constant and *Minpts* changes, *Minpts* is too small to treat the noise in the data as cluster data, and *Minpts* is too big to treat low-density data as noise. The result is that the clustering accuracy is not high, and the clustering effect is not reasonable without providing a sound decision basis and wisdom support. The clustering effect depends on the initial parameters greatly. It is an effective way to improve the clustering effect to determine the reasonable initial parameters.

DBSCAN algorithm has two significant disadvantages:

1. It is sensitive to the input parameter *Eps*, and only clusters with similar density can be found, which affects the quality of clustering results. In practice, it is challenging to determine before the algorithm is run.
2. It is challenging to find clusters with significant density differences. Because *Eps* and *MinPts* that determine the density threshold are globally unique, DBSCAN can only find clusters with approximate densities.

#### 3) Improved DBSCAN algorithm

*i*. *Clustering evaluation index based on the duty cycle*. Usually, we choose the clustering evaluation index to determine the quality of clustering results, also known as clustering validity analysis. A good clustering partition should have the following characteristics: the samples in different clusters should be as diverse as possible, and the samples in the same cluster should be as similar as possible.

It is found that the factors affecting the clustering results include not only the degree of clustering and the boundary distance between clusters but also the number of trajectory points in the cluster. Because the traditional evaluation index only considers the cohesion degree, clustering distance, and other coefficients, trajectory clustering has limitations. In the evaluation of clustering cohesion degree, the inner density of clusters is not considered, and the relationship between the number of internal clusters and the size of the cluster is ignored. In irregular clustering, the influence degree of a single variable is often too large, and the clustering result usually stays at the boundary point, which cannot achieve the optimal selection of parameters.

Aiming at the problem that the existing evaluation index is unsuitable for location point clustering, this paper proposes a novel validity index based on the internal and external duty cycle index (IEDCI). The equation is as follows:

$$r=\frac{S_{i}+S_{j}}{S_{i+j}}$$ (Eq 5)

**Fig 2:**
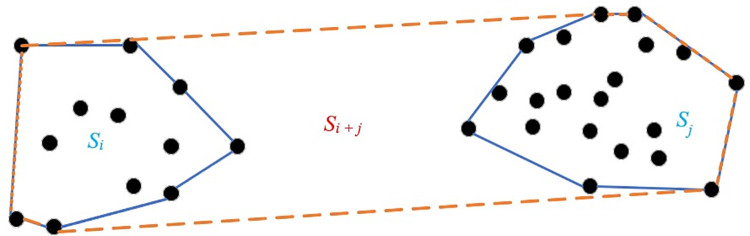
Duty cycle coefficient.

According to Eq (5), the internal and external duty cycle involves three regions *S*_*i*,_, *S*_*j*_ and *S*_*i*+*j*_ (as shown in Fig 2), which *S*_*i*_ and *S*_*j*_ are the regions enclosed by the outermost points in class *i* and *j*. *S*_*i*+*j*_ represents the region bounded by the outermost point of the two clusters merged. Duty cycle balanced relationship between the distance within the cluster and the distance between the clusters to solve the inappropriate situation of the single point being grouped or all points being grouped. The area is a two-dimensional standard, which can evaluate the degree of dispersion of two clusters, to effectively avoid the possible linear extreme distance of some points in two clusters.

After defining the internal and external duty cycle concept, an evaluation index IEDCI (Internal and External duty cycle Index) is proposed based on the internal and external duty cycle. Eq (6) is as follows:

$$F(k)=\frac{1}{k}\sum_{i=1}^{k}\underset{j\neq i}{\max}\left(\frac{\frac{n_{i}}{s_{i}}+\frac{n_{j}}{s_{j}}}{\frac{s_{i}+s_{j}}{s_{i+j}}}\right)$$ (Eq 6)

Where, *n*_*i*_、*n*_*j*_ represents the number of points in the *i*_th_ and *j*_th_ cluster, and *k* represents the number of the current clusters. $\underset{j\neq i}{Max}$ represents the maximum value of the ratio of any two different clustering sets, and *F*(*k*) is the result of the evaluation index based on the duty cycle. The smaller the value is, the better the classification result is. *F*(*k*) differences in the number of clusters may lead to different results. The clustering parameters have the best effect (cluster threshold *MinPts* and neighborhood radius *Eps*) when the value of *F*(*k*) is the smallest.

A validity evaluation index based on clustering points and the clustering duty cycle is proposed, which is used to evaluate the clustering results generated by different input parameters and determine the current optimal input parameters according to the previous feedback to find the optimal input parameters and clustering results. When processing the clustering results, the Silhouette Coefficient and the Davius-Bouldin index (DBI) only consider the relationship between the cohesion degree and the clustering distance and do not fully consider the influence of the clustering points in a single clustering result on the overall clustering effect. Therefore, the clustering evaluation proposed is better than the above evaluation indexes. The specific experimental results are shown in Tables 6 and 7.

*ii*. *A parameter-free DBSCAN algorithm*. The DBSCAN algorithm requires two critical parameters, *MinPts*, and *Eps*. However, when selecting these two clustering parameters, the DBSCAN algorithm depends on users’ practical experience. The improved DBSCAN clustering algorithm proposed clusters the data and automatically determines the input parameters: the input parameters of the evaluation index are the clustering results generated during the clustering process, and then the evaluation results are obtained. The specific algorithm is shown in Table 1.

**Table 1:** Improved DBSCAN clustering algorithm.

**Input: *D* = {D**_*1*_ *(x*_1_, *y*_1_*)*, *D*_2_ *(x*_2_, *y*_2_*)*,.* *.* *. *D*_*m*_ *(x*_m_, *y*_m_*)}* ***E*, *M*** *MaxNum*, *MinNum*, *MaxEps*, *MinEps* **Output: *resultC*, *MinIDECI*, *bestEps*, *bestMinPts***
1. Set ***D*** = undefined 2. **for** *Eps* in ***E*** and *Minpts* in ***M* do** 3. **for** each *p* in ***D* do** 4. **If** label(*p*) ≠ undefined **then** 5. continue **6.** **end** 7. **else** 8. Check *Eps(p)* **if** *Eps(p)* < *MinPts* **then** 9. The Label (*p*) = Noise; 10. **end** **11.** **else** 12. Label*(p) =* core, new *C*, *Eps(p)* join ***C*** **for** *q* in *Eps(p)* and Label(*q*) = undefined **do** 13. Check *Eps(q)* if *Eps(q)* and *Label(q)* not in any **C then** 14. *Eps(q)* join *C* **15.** **end** **16.** **end** **17.** **end** **18.** **end** **19.** **end** 20. if *IEDCI(C)* < *MinIDECI* **then** 21. *MinIDECI* = *IDECI(C)* 22. *bestEps* = *Eps* 23. *bestMinPts* = *MinPts* 24. *resultC* = C 25. Eps + = 1,MinPts + = 1 **26.** **end** **27.** **end**

1. **The input parameters**
  ■ ***D*** is the current input data set. ***D***_**1**_ (*x*_1_, *y*_1_) represents *x* and *y* of the plane coordinates in the set.
  ■ *MaxEps* is the maximum distance between two plane coordinate points, which can be flexibly determined according to practical meaning.
  ■ *MinEps* is the minimum distance between two plane coordinate points, which can be flexibly determined according to practical meaning.
  ■ E is the distance between any two points in the set, ranging from *MinEps* to *MaxEps*.
  ■ *MaxNum* sets the upper limit of the clustering threshold because if the number of clusters is too large, the data set may not cluster effectively.
  ■ *MinNum* sets the lower limit of the cluster threshold. If the number of clusters is too small, it may lead to too many clusters, and even a point becomes a cluster without a calculated value.
  ■ M determines the optimal quantity threshold for a cluster, which ranges between *MaxNum* and *MinNum*.
2. **Output parameters**
  ■ *ResultC* is the clustering result. Different input parameters can be used to get different clustering results.
  ■ *MinIEDCI* is the minimum duty cycle and is initially set to infinity.
  ■ *BestEps* is the best value for **E**, initially set to 0.
  ■ *Best MinPts* is the best value for **M**, with an initial value of 0.

Different input parameters will produce different clustering results. The algorithm gives a range of input parameters, traverses all parameter values within the field, and generates clustering results to prevent some parameters from being lost. The optimal evaluation value can be obtained by evaluating and calculating the clustering results, and the optimal input parameters can be calculated based on the backpropagation method. The algorithm process is described as follows:

**STEP 1 Building the input parameter range**

In different application scenarios, the value of the optimal clustering input parameter fluctuates within a specific range. Since the range of input parameters determines the efficiency of algorithm execution and the possibility of finding the optimal value, it is essential to establish a suitable range of input parameters before algorithm execution. Too many clustering times, the data set may not form effective clustering; Too few clustering times and too scattered clustering are not practical. In addition, the distance between cluster points will affect the compactness within the cluster. If the distance measure is too large, the clusters are too discrete to distinguish different clusters effectively. If the distance measure is too small, the clustering distance is too close, producing too many trivial and worthless clustering results. Therefore, at the early stage of clustering, the maximum and minimum values of *Eps* and *MinPts* should be determined to construct the effective range of clustering parameters.

**STEP 2 Generating clustering results**

Cyclic density clustering was carried out to complete the clustering calculation of all travelers’ trajectory points for six months using the neighborhood radius range in Step 1 as the input parameter. All the clustering results (result ***C***) were saved.

**STEP 3 Evaluating the clustering results**

Evaluation indexes such as Silhouette Coefficient, DBI (David-Bouldin index), and IEDCI (Internal and External Duty Cycle Index) were used to evaluate the clustering results. The best clustering parameters *BestEps* and *BestMinPts* were saved in the evaluation index.

**STEP 4 Obtaining the optimal clustering results**

The best clustering result was calculated, taking *BestEps* and *BestMinPts* in Step 3 as input parameters. The clustering result is the clustering of the actual activity trajectory of the travelers, which is all the possible travel areas of the public transport travelers in the follow-up research.

### B. Data preprocessing

#### 1) Acquisition of travelers’ trajectory data

In this study, the trajectory points of individual users’ smartphones were collected as the original data set. The GPS positioning information was mainly obtained using the integrated positioning sensor in the experimental mobile phone for recording and storage. At present, Baidu Map, Tencent Map, and Google Map all provide map-based API development applications. With the authorization of smartphone users, the collection of smartphone users’ trajectory points can be realized by using the APP of mobile phones. According to the setting of the positioning software, the user’s trajectory point information is collected every 10 seconds. In an ideal state, the number of trajectory points collected in a day should be 8640. However, the number of recorded trajectory points varies under different positioning strategies due to the differences in hardware performance, operating systems, carrier networks, and signal stability of location sensors of users’ smartphones.

#### 2) Preprocessing of travelers’ trajectory data

It is necessary to preprocess the collected original trajectory point data to meet the data format requirements of the clustering algorithm. In this paper, the GPS coordinate data processed by Sage-Husa adaptive filtering is preprocessed again, including data cleaning, data centralization, and standardization. Data cleaning aims to eliminate null values in the original data set, remove or correct incorrect data, and prepare for the accurate discovery of clustering patterns. Data centralization and standardization is since all variables in the collected data often use different units of measurement, and the difference between the data is significant. The data with a large absolute value can overwhelm a small absolute value in the clustering process. The latter cannot reflect the characteristics of the existing system.

The accuracy of the GPS is easily affected by environmental factors. Therefore, taking location L_1_ (downtown) and location L_2_(remote valley), the GPS integrated device is used to output positioning data every 5s for a fixed location. The 500 continuously acquired data are composed of the original data set algorithm test.

The data distribution after Sage-Husa adaptive filtering, the mean error of longitude and latitude of L_1_ is 4.6 m. The mean error of longitude and latitude of L_2_ is 6.3m (as shown in Table 2). After calculation, the proposed algorithm is close to the actual coordinate and meets the GPS positioning error of 3–5 m.

**Table 2:** Comparison of positioning errors between L_1_ and L_2_.

Location	Longitude (°)	Latitude (°)	Error (m)
Measured Coordinates of L_1_ (Jinmao Tower)	120.097832	29.324656	2.1
Actual coordinates of L_1_	120.097858	29.324661	
Measured Coordinates of L_2_ (The Double Dragon Cave Visitor Centre)	119.622086	29.201382	2.5
Actual coordinates of L_2_	119.622114	29.201413	

### C. Regional clustering of public transport travel

**Input:** preprocessed mobile phone GPS data set (site demand set) ***D***, accessibility reference set ***S*** including bus stops, subway stops, and other transportation hubs.

**Output:** the urban public transport travel area (set of alternative stops) generated based on the input data.

**Steps:**

1. Set the set ***D*** of access requirement points ***D***_***w***_ ***= D*,** cluster number ***k = 0*,** cluster partition set ***C***_***rc***_ = Ø;
2. Perform the parameter-free DBSCAN algorithm in Table 1.
3. The clustering result is *C*_***k***_, the points which be divided into *C*_***k***_ will be removed from the set *D*_***w***_;
4. If the D_***w***_ = Ø, then output the clustering result as the location of the alternative site, C_***rc***_ = {***C***_**1**_**, *C***_**2**_**…, *C***_***k***_}, otherwise, go back to step (2) again;

Using the improved parameter-free DBSCAN algorithm to solve the location problem of original-destination of public transport, we can get the travel area of public transport (alternative set of stations) **Site = *S*∪*C***_***rc***_ = **{*S***_**1**_**, *S***_**2**_**,…,*S***_***i***_**,…*S***_***N1***_**, *C***_**1**_**, *C***_**2**_**,…, *C***_***j***_**,…, *C***_***N2***_**}.**

## V. Case study

### A. Data set

#### 1) 2D synthetic data sets

**Fig 3:**
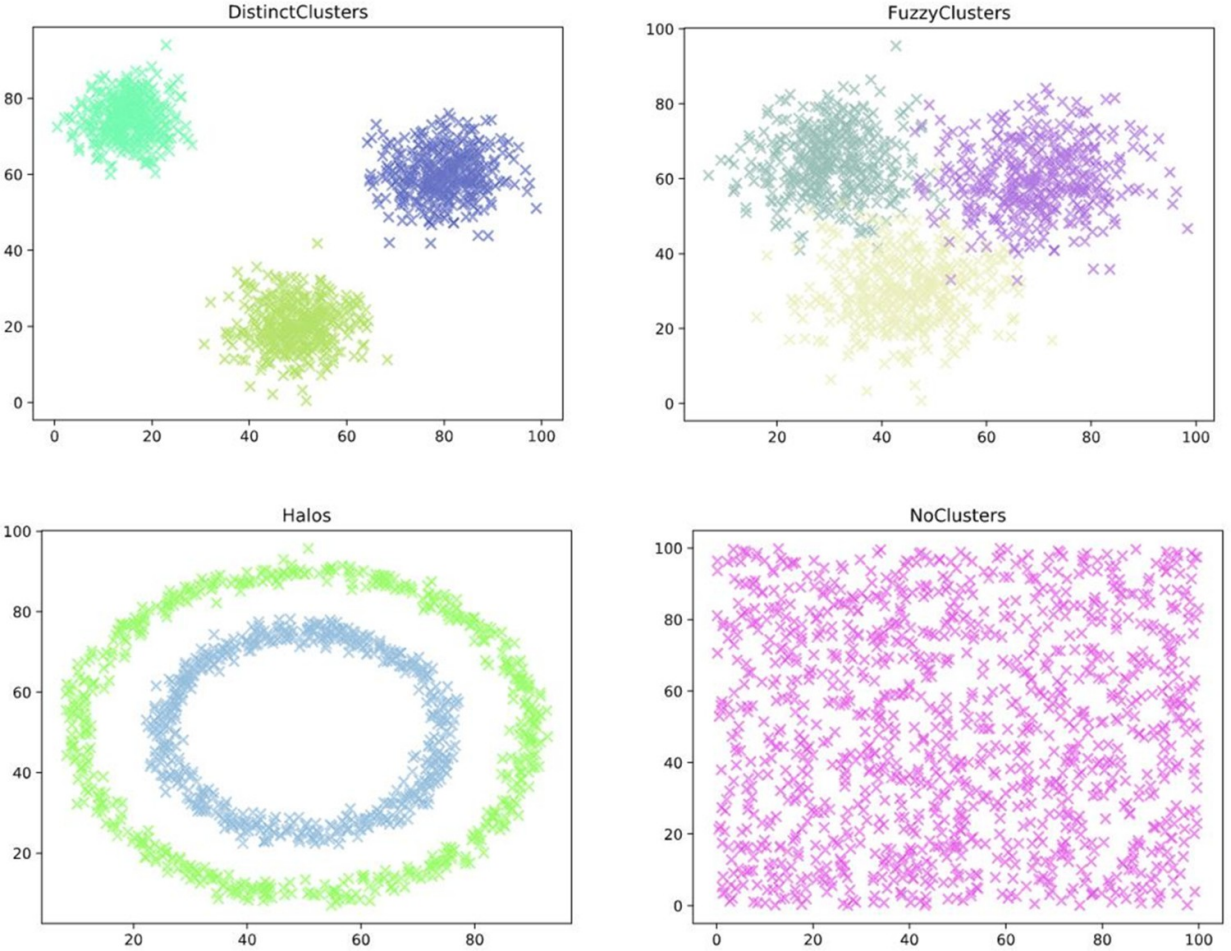
2-D synthetic data sets.

2D synthetic data sets included random numbers generated for a computer simulation. Each data set has 1,200 points, each represented as a coordinate and divided into a cluster. These data sets are Distinct Cluster, Fuzzy cluster, Halo cluster, and non-cluster (as shown in Fig 3). In these data sets, the structure of the clear cluster and fuzzy cluster are convex, the design of the halo cluster is annular, and the form of non-cluster is splash.

#### 2) Case data set

The case data used in this paper are from the ebus APP, which is about urban public transport mobile software. Travelers can use ebus to query public transportation information such as nearby stations, line transfers, real-time arrival prediction. During the query process, the user’s location information is synchronously recorded in the ebus APP. This paper used the location information data of 500 volunteers in Jinhua city over the past six months (From January 2021 to June 2021 (see S2 File)). A total of 500. TXT files were obtained, each representing each traveler’s location information during the six months. The trajectory data of each traveler is represented by the x, y coordinates of the trajectory points. In addition, since the data set represents the real traveler’s trajectory points, the data structure is varied compared to the 2D synthetic data sets, including linear, annular, convex, and splash. The case data set is shown in Table 3, where UID is the unique identifier of the user SIM card, LNG is the longitude of the current user position, LAT is the dimension of the current user position, and UP_TIME is the coordinate upload time (see Table 1 in S1 File).

**Table 3:** The travel data structure of urban public transport in Jinhua city.

	UID	LNG	LAT	UP_TIME
1	3615691134	119.666871	29.068345	02/01/2021 11:23:56
2	3286093069	119.661293	29.070279	02/10/2021 08:01:12
3	1778287686	119.658382	29.072213	02/23/2021 20:17:04
4	4189128205	119.663557	29.076293	02/27/2021 18:43:33
5	. . .	. . .	. . .	. . .

To verify the reliability and accuracy of the Sage-Husa-based adaptive filtering algorithm proposed, the GPS data collected is de-noised and compared with the original GPS data without denoising. Table 4 is the comparison and analysis results of the positioning errors of the algorithm in different directions and dimensions.

**Table 4:** Comparison of positioning accuracy based on Sage-Husa adaptive filtering algorithm.

Direction	Eastward	Northward	2-D dimensional	3-D dimensional
Data processed by the algorithm (m)	4.72	5.45	6.56	9.73
Raw GPS Data (m)	5.71	6.43	7.65	11.89
Precision improvement ratio (%)	17.34%	15.24%	14.25%	18.17%

The case shows that the proposed algorithm effectively controls the error range and further improves the positioning accuracy of GPS. Positioning accuracy eastward and northward are improved by 17.34% and 15.24%, respectively, and the positioning accuracy in 2D and 3D dimensions is enhanced by 14.25% and 18.17%, respectively. After using this algorithm, the number of noise points is reduced obviously, and the data error is controlled in a smaller range and concentrated to the actual coordinate points.

### B. Comparison on parameter selection

In traveler trajectory mining, *Eps* is the walking distance of the traveler, and *MinPts* is the number of the dwelling of traveler in a specific area, both of which have practical significance. Therefore, the parameter range can be delimited according to the real meaning. According to the statistics of existing data, it is concluded that the walking radius of travelers is mostly between 20 meters and 110 meters. Therefore, the *Eps* threshold was set within (20,110) in the experiment. All subsequent experimental tests are based on this range.

Clustering with too few or too many points is of no practical significance because too small a clustering coordinate threshold may be a noise point, and it is not easy to find the clustering with a large threshold. The threshold value of *MinPts* was set within (8,13), and subsequent experimental tests were based on this range.

The case data set was used, and the clustering results were generated and compared with other parameters, including empirical and statistical values, to verify the performance of the parameters automatically selected by the improved DBSCAN algorithm.

**Fig 4:**
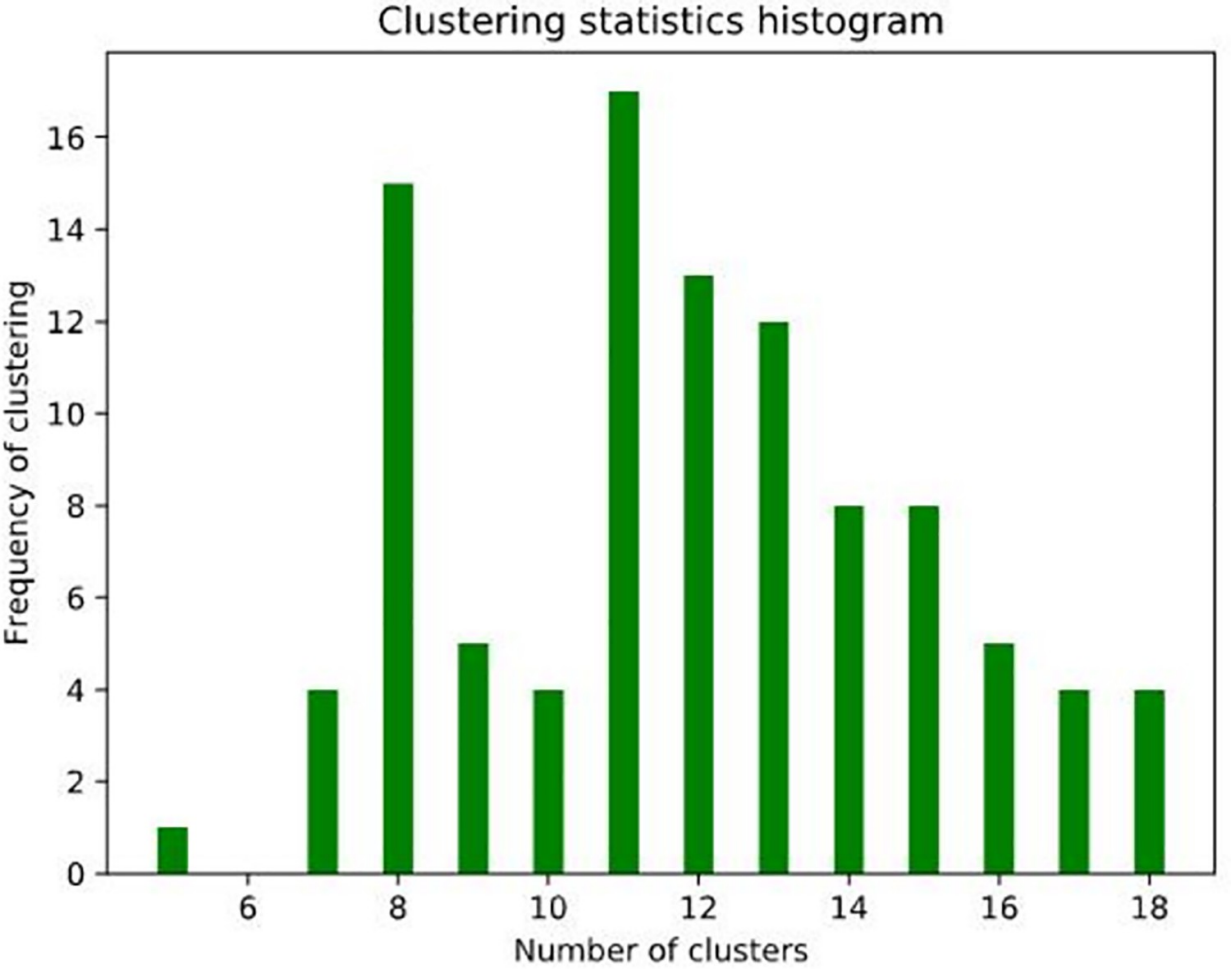
Frequency of clustering results.

The results for all input parameters are counted to find the most common cluster number (see Fig 4). Statistically analyze the input parameters at this point, the median of the current input parameters (60,12) is taken as the statistical input parameters (*Eps* value is 60, *MinPts* value is 12). The *Eps* and *MinPts* values obtained from experience were 85 and 10, respectively. The *Eps* and *MinPts* obtained by improved DBSCAN were 65 and 12, respectively.

The case data set contains 500 individual location points. Compactness, separation, and DBI were used to evaluate the clustering results. Compactness and DBI represent the degree of cohesion of the clusters, and the degree of separation represents the distance between the clusters. The smaller the compactness and DBI, the higher the separation value, and the better the clustering effect. As shown in Table 5, the parameters automatically generated by the method proposed in this paper achieved a better clustering effect in terms of separation degree and DBI. Compared with the traditional experience value, the performance of this method has been dramatically improved.

**Table 5:** Experimental results of different performance parameters.

Input parameters	Experience value	Statistics	Improved DBSCAN
**(Eps, MinPts)**	**(85, 10)**	**(60, 12)**	**(65, 12)**
Compactness	4.373	**3.086**	4.047
Separation	588.469	568.858	**597.962**
Davius-Bouldin index (**DBI**)	0.129	0.992	**0.089**

### C. Comparison of evaluation indexes

2D synthetic data sets and case data set respectively are used to generate clustering results and compare them with other validity indicators, including DBI and Silhouette Coefficient evaluation, to verify the performance of IEDCI.

#### 1) 2D synthetic data sets

This paper uses compactness and separation to evaluate the clustering results of four 2-D synthetic data sets. Table 6 shows the compactness evaluation results of the three evaluation indexes. According to the results, IEDCI has a better evaluation value for the distinct cluster, fuzzy cluster, and non-cluster data set. Table 7 shows the separation evaluation results of the three evaluation indexes. It can be seen from the results that IEDCI has a better evaluation value for clear cluster and non-cluster data sets.

**Table 6:** Compactness evaluation results of different performance indexes.

The evaluation index	Silhouette Coefficient	DBI	IEDCI
Distinct cluster	73287.920	69802.893	**67994.399**
Fuzzy cluster	130473.570	85870.576	**53500.792**
Halos Clusters	324076.380	**7008.825**	324113.390
No-cluster	33617.257	20486.406	**20397.885**

**Table 7:** Separation evaluation results of different performance indexes.

The evaluation index	Silhouette Coefficient	DBI	IEDCI
Distinct cluster	602379.301	600588.843	**610914.479**
Fuzzy cluster	234766.280	**492982.838**	259148.034
Halos Clusters	**25905.327**	188408.806	25876.882
No-cluster	539210.783	3836.680	**571323.930**

#### 2) Case data set

Case data sets are to be used to evaluate the performance of the algorithm. The entire evaluation process is as follows.

**Fig 5:**
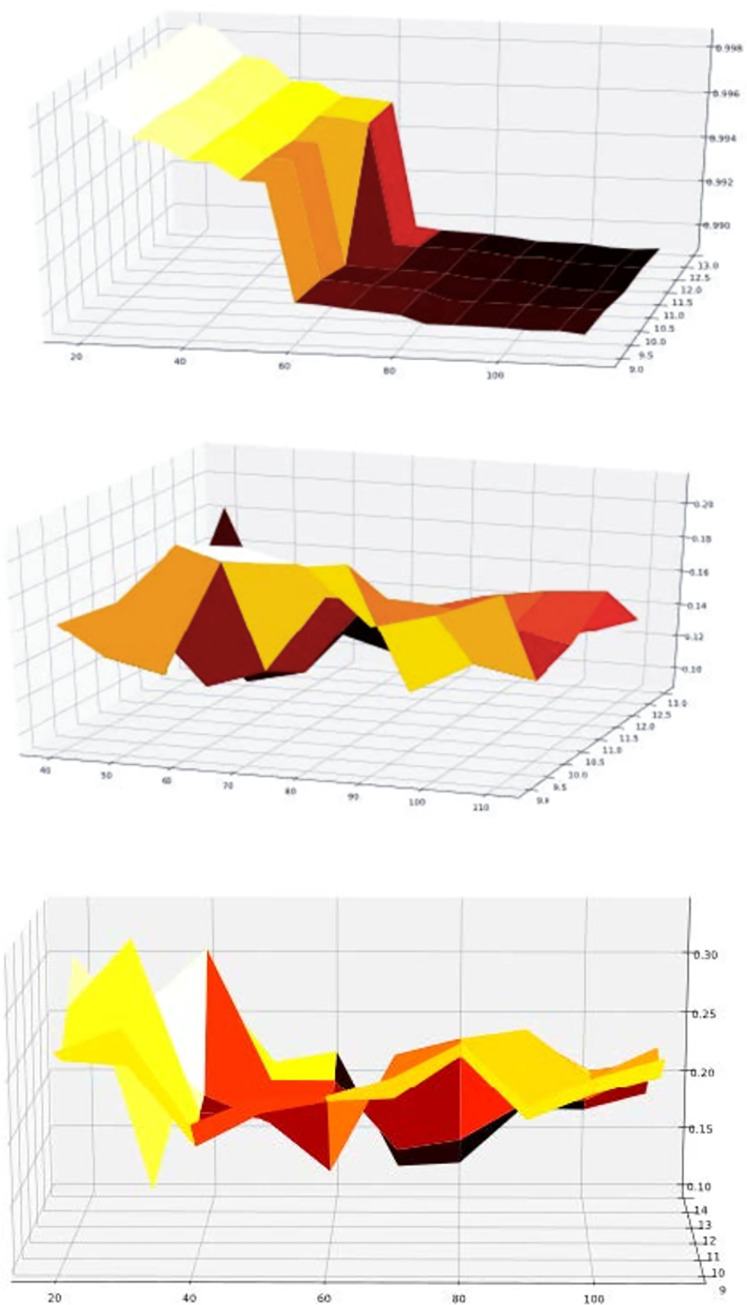
3-Dimensional surfaces with different performance indexes.

**(1) Optimal input selection:** The algorithm in Table 1 was performed using the three evaluation indexes of Silhouette Coefficient, DBI, and IEDCI. After traversing all possible values in the parameter range, the algorithm can obtain the optimal input parameters corresponding to the three evaluation indexes, as shown in Table 8. We use a three-dimensional surface diagram to illustrate the process of getting the optimal input parameters (see Fig 5).

**Table 8:** Optimal values of *MinPts* and *Eps*.

The evaluation index	Silhouette Coefficient	DBI	IEDCI
**Best *MinPts* value**	13	12	14
**Best *Eps* value**	10	50	30

In the figure, the axis *x* represents all possible values of *Eps*, the coordinate *y* represents all possible values of *MinPts*, and the coordinate *z* represents the corresponding values. The values of *Eps* and *MinPts* are the best when the value is the smallest. After parameter selection within the same input range, the optimal parameter value generated by the Silhouette Coefficient will be generated at the boundary point, and both DBI and IEDCI will obtain the optimal parameter value within the range, minimize the value of the evaluation index.

**Fig 6:**
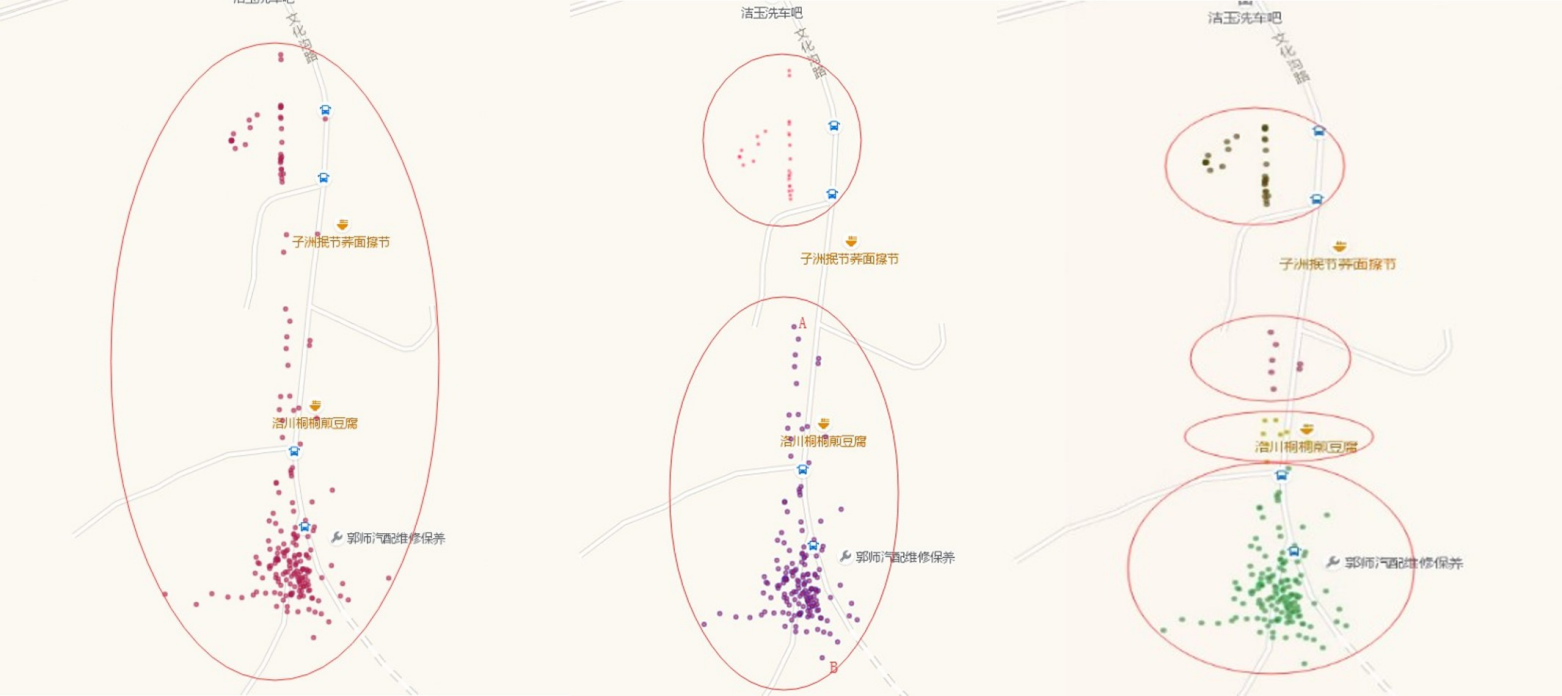
Clustering results of different performance indicators.

**(2) Clustering results:** Three different clustering results are generated using the optimal input values of the three evaluation indexes. As shown from Fig 6, for the clustering points within the same range, the results generated by the evaluation index of the Silhouette Coefficient gather the discrete points in the red ellipse into a cluster. However, from the actual situation of the traveler’s trajectory, the clustering results are unsatisfactory because travelers have too many activities. In the DBI clustering results, the red ellipse is divided into two parts. Similarly, the distance from point A to point B in Fig 6(b) is far beyond the range of human activities (500 meters). In this paper’s clustering results of the algorithm, the range of traveler’s movement is smaller than the radius of the resident’s trajectory. Therefore, this algorithm performs well in practical applications.

**(3) Clustering evaluation:** The compactness and separation degree of the generated clustering results were evaluated, and the evaluation results are shown in Table 9. Based on fully considering the effects of clustering density and clustering spacing, the results obtained by the proposed method have bigger separation and smaller compactness, which is more consistent with the actual situation of human activities in trajectory clustering.

**Table 9:** Evaluation results of separation and compactness.

Evaluation index	Silhouette Coefficient	DBI	IEDCI
Compactness	6.430	2.804	**1.647**
Separation	639.290	567.494	**653.907**

## VI Conclusion

This study aims to explore the extraction scheme about spatial dwell point suitable for mobile intelligent terminal users’ trajectory.

1. A more accurate, efficient, and noise-resistant mobile phone GPS data preprocessing method makes it possible to efficiently mine travel trajectory data. The Sage -Husa adaptive filtering algorithm can effectively determine the error range and further improve the GPS positioning accuracy: the positioning accuracy in the eastward and northward is improved by 17.34% and 15.24%, respectively, and the positioning accuracy in 2-D and 3-D dimensions are improved by 14.25% and 18.17%, respectively. After using this algorithm, the number of noise points is reduced obviously. The GPS of smartphones can improve the positioning accuracy through the proposed adaptive filtering algorithm, which has the advantages of low cost and convenient use and can meet the positioning accuracy requirements for DBSCAN spatial clustering.
2. This paper puts forward a novel traveler trajectory mining method, using a novel evaluation index to evaluate the input parameters of DBSCAN. The evaluation index balances the distance within the cluster and the distance between the clusters to obtain the optimal input parameters of location data clustering. The problem of parameter inaccuracy caused by manual experience is avoided. Secondly, based on the travel data of Jinhua city, the method proposed is verified, and the experiment shows that the algorithm proposed can find the optimal input parameter value on the traveler trajectory data set. By calculating the compactness and separation of the clustering results, it is found that compared with the Davies-Bouldin index (DBI) and Silhouette Coefficient, the optimal parameter value found by IEDCI has a smaller cohesion value and a larger cluster spacing value. Therefore, the proposed algorithm has good performance in the regional clustering of public transport travelers.

Based on the above two points, the reliability of identification results can be significantly improved. However, at the same time, there are also the following problems:

1. Due to time constraints and privacy considerations, recruiting volunteers for mobile positioning data is difficult, so the data amount is small, and large-scale trajectory data mining and verification cannot be carried out.
2. This paper does not consider the accuracy and reliability of mobile phone GPS single-point positioning in complex and harsh environments.
3. Some outliers in the initial data set have not been deeply analyzed. Improving the quality of track point acquisition is the prerequisite for the clustering algorithm’s accuracy of dwell point extraction. From the perspective of the clustering algorithm, it remains further discussed to study the time interval, missing data collection, and positioning error of trajectory points.

Due to the particularity of data and the diversity and complexity of requirements, future research should focus on the following points:

1. Increase the amount of mobile phone positioning data. It is hoped that more mobile phone trajectory data can be obtained in the next step to carry out large-scale trajectory data mining and verification.
2. An in-depth study on the mobile intelligent terminal development model and trajectory data acquisition, data preprocessing, and WebGIS system integration should be carried out. Form a more automatic and intelligent trajectory extraction application about dwell points in mobile phone APP.
3. Big data is currently a hot topic. However, it is also a huge challenge, on the one hand, provides researchers with the data source, on the other hand, also asked to select the effective data, based on guarantee the clustering effect, and explore a suitable, lower time complexity, higher execution efficiency of the extraction and clustering algorithm of massive Spatio-temporal dwell point trajectory,
4. Conduct sufficient research on trajectory data pattern mining and personal travel feature analysis. In this paper, the travel mode, travel distance, time period, trajectory semantics, and other issues are combined to explore the valuable potential information contained in the trajectory data of smartphone users by using dwell point data.
5. Promotion and application: The proposed method can be used to cluster the location information of travelers to obtain the original-destination of travel and be extended to logistics and supply chain management, vehicle dynamic routing, gas station planning, and other routing problems. That is because all these problems are point clustering problems on two-dimensional maps. Unlike other clusters, the size of a cluster is limited because people or vehicles have a specific range of movement.

## Supporting information

S1 File Data structure. (DOCX)

S2 File The layout of Jinhua city transit network (2020). (DOCX)
