# Supplementary material for: Research on the method of travel area clustering of urban public transport based on Sage-Husa adaptive filter and improved DBSCAN algorithm
Source: PLoS One. 2021 Dec 22;16(12):e0259472. doi: 10.1371/journal.pone.0259472 (PMC8694428; doi:10.1371/journal.pone.0259472)
Supplement: S1 File — (DOCX) [file pone.0259472.s001.docx]

Supporting information

**S1. Data structure**

**Table 1. A passenger's** [**trajectory**](javascript:;) **record in April 2021(UID:** 1742800653**)**

| **UID** | **LNG** | **LAT** | **UP_TIME** | **Location** |
| --- | --- | --- | --- | --- |
| 1742800653 | 119.638016 | 29.128459 | 04/01/2021 07:03:56 | ZJNU |
| 1742800653 | 119.666871 | 29.068345 | 04/01/2021 07:57:07 | South Bus hub terminus |
| 1742800653 | 119.666871 | 29.068345 | 04/01/2021 14:03:58 | South Bus hub terminus |
| 1742800653 | 119.663557 | 29.076293 | 04/01/2021 14:15:40 | Traffic Police First squadron |
| 1742800653 | 119.663557 | 29.076293 | 04/01/2021 16:33:04 | Traffic Police First squadron |
| 1742800653 | 119.638016 | 29.128459 | 04/01/2021 17:20:51 | ZJNU |
| 1742800653 | 119.638016 | 29.128459 | 04/02/2021 07:01:21 | ZJNU |
| 1742800653 | 119.666871 | 29.068345 | 04/02/2021 07:45:02 | South Bus hub terminus |
| 1742800653 | 119.666871 | 29.068345 | 04/02/2021 17:03:22 | South Bus hub terminus |
| 1742800653 | 119.638016 | 29.128459 | 04/02/2021 17:58:24 | ZJNU |
| 1742800653 | 119.638016 | 29.128459 | 04/03/2021 07:01:24 | ZJNU |
| 1742800653 | 119.666871 | 29.068345 | 04/03/2021 07:55:24 | South Bus hub terminus |
| 1742800653 | 119.666871 | 29.068345 | 04/03/2021 16:03:16 | South Bus hub terminus |
| 1742800653 | 119.653283 | 29.101673 | 04/03/2021 16:35:29 | Insein square |
| 1742800653 | 119.653283 | 29.101673 | 04/03/2021 18:43:24 | Insein square |
| 1742800653 | 119.638016 | 29.128459 | 04/03/2021 19:25:09 | ZJNU |
| 1742800653 | 119.638016 | 29.128459 | 04/04/2021 09:03:24 | ZJNU |
| 1742800653 | 119.653573 | 29.116807 | 04/04/2021 09:23:07 | Jinhua Public Security Bureau |
| 1742800653 | 119.653573 | 29.116807 | 04/04/2021 10:03:58 | Jinhua Public Security Bureau |
| 1742800653 | 119.649346 | 29.083761 | 04/04/2021 10:56:40 | Jiangnan Fu Tai long |
| 1742800653 | 119.649346 | 29.083761 | 04/04/2021 17:43:27 | Jiangnan Fu Tai long |
| 1742800653 | 119.638016 | 29.128459 | 04/04/2021 18:35:21 | ZJNU |
| 1742800653 | 119.638016 | 29.128459 | 04/05/2021 11:23:56 | ZJNU |
| 1742800653 | 119.650832 | 29.103416 | 04/05/2021 11:55:07 | Huanyan building |
| 1742800653 | 119.650832 | 29.103416 | 04/05/2021 13:03:58 | Huanyan building |
| 1742800653 | 119.638016 | 29.128459 | 04/05/2021 13:20:51 | ZJNU |
| 1742800653 | 119.638016 | 29.128459 | 04/06/2021 07:02:15 | ZJNU |
| 1742800653 | 119.666871 | 29.068345 | 04/06/2021 07:57:07 | South Bus hub terminus |
| 1742800653 | 119.666871 | 29.068345 | 04/06/2021 16:50:15 | South Bus hub terminus |
| 1742800653 | 119.638016 | 29.128459 | 04/06/2021 17:20:51 | ZJNU |
| 1742800653 | 119.638016 | 29.128459 | 04/07/2021 07:10:56 | ZJNU |
| 1742800653 | 119.666871 | 29.068345 | 04/07/2021 08:05:23 | South Bus hub terminus |
| 1742800653 | 119.666871 | 29.068345 | 04/07/2021 16:03:33 | South Bus hub terminus |
| 1742800653 | 119.653573 | 29.116807 | 04/07/2021 16:55:35 | Jinhua Public Security Bureau |
| 1742800653 | 119.653573 | 29.116807 | 04/07/2021 17:33:04 | Jinhua Public Security Bureau |
| 1742800653 | 119.638016 | 29.128459 | 04/07/2021 17:58:11 | ZJNU |
| 1742800653 | 119.638016 | 29.128459 | 04/08/2021 07:00:26 | ZJNU |
| 1742800653 | 119.666871 | 29.068345 | 04/08/2021 07:50:07 | South Bus hub terminus |
| 1742800653 | 119.666871 | 29.068345 | 04/08/2021 16:43:28 | South Bus hub terminus |
| 1742800653 | 119.638016 | 29.128459 | 04/08/2021 17:20:51 | ZJNU |
| 1742800653 | 119.638016 | 29.128459 | 04/09/2021 07:00:24 | ZJNU |
| 1742800653 | 119.666871 | 29.068345 | 04/09/2021 07:54:05 | South Bus hub terminus |
| 1742800653 | 119.666871 | 29.068345 | 04/09/2021 17:03:43 | South Bus hub terminus |
| 1742800653 | 119.638016 | 29.128459 | 04/09/2021 17:53:51 | ZJNU |
| 1742800653 | 119.638016 | 29.128459 | 04/10/2021 09:23:56 | ZJNU |
| 1742800653 | 119.653573 | 29.116807 | 04/10/2021 09:53:02 | Jinhua Public Security Bureau |
| 1742800653 | 119.653573 | 29.116807 | 04/10/2021 13:03:58 | Jinhua Public Security Bureau |
| 1742800653 | 119.638016 | 29.128459 | 04/10/2021 13:20:54 | ZJNU |
| 1742800653 | 119.638016 | 29.128459 | 04/11/2021 07:50:22 | ZJNU |
| 1742800653 | 119.658251 | 29.121160 | 04/11/2021 07:57:07 | A18 |
| 1742800653 | 119.658251 | 29.121160 | 04/11/2021 11:33:23 | A18 |
| 1742800653 | 119.638016 | 29.128459 | 04/11/2021 11:40:21 | ZJNU |
| 1742800653 | 119.638016 | 29.128459 | 04/12/2021 07:11:56 | ZJNU |
| 1742800653 | 119.666871 | 29.068345 | 04/12/2021 07:50:07 | South Bus hub terminus |
| 1742800653 | 119.666871 | 29.068345 | 04/12/2021 17:03:58 | South Bus hub terminus |
| 1742800653 | 119.638016 | 29.128459 | 04/12/2021 18:10:23 | ZJNU |
| 1742800653 | 119.638016 | 29.128459 | 04/13/2021 07:13:56 | ZJNU |
| 1742800653 | 119.666871 | 29.068345 | 04/13/2021 08:07:07 | South Bus hub terminus |
| 1742800653 | 119.666871 | 29.068345 | 04/13/2021 16:03:58 | South Bus hub terminus |
| 1742800653 | 119.656207 | 29.103121 | 04/13/2021 16:55:10 | Lotus tower |
| 1742800653 | 119.656207 | 29.103121 | 04/13/2021 18:33:16 | Lotus tower |
| 1742800653 | 119.638016 | 29.128459 | 04/13/2021 19:20:32 | ZJNU |
| 1742800653 | 119.638016 | 29.128459 | 04/14/2021 07:00:27 | ZJNU |
| 1742800653 | 119.666871 | 29.068345 | 04/14/2021 07:50:31 | South Bus hub terminus |
| 1742800653 | 119.666871 | 29.068345 | 04/14/2021 17:01:21 | South Bus hub terminus |
| 1742800653 | 119.638016 | 29.128459 | 04/14/2021 18:10:47 | ZJNU |
| 1742800653 | 119.638016 | 29.128459 | 04/15/2021 07:13:56 | ZJNU |
| 1742800653 | 119.666871 | 29.068345 | 04/15/2021 07:57:07 | South Bus hub terminus |
| 1742800653 | 119.666871 | 29.068345 | 04/15/2021 17:00:31 | South Bus hub terminus |
| 1742800653 | 119.638016 | 29.128459 | 04/15/2021 17:50:29 | ZJNU |
| 1742800653 | 119.638016 | 29.128459 | 04/16/2021 07:09:23 | ZJNU |
| 1742800653 | 119.666871 | 29.068345 | 04/16/2021 07:57:07 | South Bus hub terminus |
| 1742800653 | 119.666871 | 29.068345 | 04/16/2021 15:23:21 | South Bus hub terminus |
| 1742800653 | 119.663557 | 29.076293 | 04/16/2021 15:35:40 | Traffic Police First squadron |
| 1742800653 | 119.663557 | 29.076293 | 04/16/2021 16:20:04 | Traffic Police First squadron |
| 1742800653 | 119.638016 | 29.128459 | 04/16/2021 17:10:18 | ZJNU |
| 1742800653 | 119.638016 | 29.128459 | 04/17/2021 09:13:22 | ZJNU |
| 1742800653 | 119.653283 | 29.101673 | 04/17/2021 09:47:09 | Insein square |
| 1742800653 | 119.653283 | 29.101673 | 04/17/2021 18:11:43 | Insein square |
| 1742800653 | 119.638016 | 29.128459 | 04/17/2021 18:59:03 | ZJNU |
| 1742800653 | 119.638016 | 29.128459 | 04/18/2021 09:13:56 | ZJNU |
| 1742800653 | 119.654909 | 29.080843 | 04/18/2021 09:47:07 | South Bayi Street-Li Yu Road |
| 1742800653 | 119.654909 | 29.080843 | 04/18/2021 09:53:11 | South Bayi Street-Li Yu Road |
| 1742800653 | 119.677890 | 29.089581 | 04/18/2021 10:15:40 | Wanda plaza |
| 1742800653 | 119.677890 | 29.089581 | 04/18/2021 18:33:04 | Wanda plaza |
| 1742800653 | 119.654909 | 29.080843 | 04/18/2021 18:53:05 | South Bayi Street-Li Yu Road |
| 1742800653 | 119.654909 | 29.080843 | 04/18/2021 19:03:39 | South Bayi Street-Li Yu Road |
| 1742800653 | 119.638016 | 29.128459 | 04/18/2021 19:52:51 | ZJNU |
| 1742800653 | 119.638016 | 29.128459 | 04/19/2021 07:00:56 | ZJNU |
| 1742800653 | 119.666871 | 29.068345 | 04/19/2021 07:53:21 | South Bus hub terminus |
| 1742800653 | 119.666871 | 29.068345 | 04/19/2021 17:03:58 | South Bus hub terminus |
| 1742800653 | 119.638016 | 29.128459 | 04/19/2021 17:57:51 | ZJNU |
| 1742800653 | 119.638016 | 29.128459 | 04/20/2021 07:10:11 | ZJNU |
| 1742800653 | 119.666871 | 29.068345 | 04/20/2021 08:07:22 | South Bus hub terminus |
| 1742800653 | 119.666871 | 29.068345 | 04/20/2021 16:53:45 | South Bus hub terminus |
| 1742800653 | 119.638016 | 29.128459 | 04/20/2021 17:40:24 | ZJNU |
| 1742800653 | 119.638016 | 29.128459 | 04/21/2021 07:01:16 | ZJNU |
| 1742800653 | 119.666871 | 29.068345 | 04/21/2021 07:55:17 | South Bus hub terminus |
| 1742800653 | 119.666871 | 29.068345 | 04/21/2021 14:23:58 | South Bus hub terminus |
| 1742800653 | 119.663557 | 29.076293 | 04/21/2021 14:35:40 | Traffic Police First squadron |
| 1742800653 | 119.663557 | 29.076293 | 04/21/2021 16:58:04 | Traffic Police First squadron |
| 1742800653 | 119.638016 | 29.128459 | 04/21/2021 17:27:51 | ZJNU |
| 1742800653 | 119.638016 | 29.128459 | 04/22/2021 06:58:21 | ZJNU |
| 1742800653 | 119.666871 | 29.068345 | 04/22/2021 07:45:13 | South Bus hub terminus |
| 1742800653 | 119.666871 | 29.068345 | 04/22/2021 13:33:58 | South Bus hub terminus |
| 1742800653 | 119.663557 | 29.076293 | 04/22/2021 13:55:40 | Traffic Police First squadron |
| 1742800653 | 119.663557 | 29.076293 | 04/22/2021 16:44:21 | Traffic Police First squadron |
| 1742800653 | 119.638016 | 29.128459 | 04/22/2021 17:40:43 | ZJNU |
| 1742800653 | 119.638016 | 29.128459 | 04/23/2021 07:10:06 | ZJNU |
| 1742800653 | 119.666871 | 29.068345 | 04/23/2021 07:58:43 | South Bus hub terminus |
| 1742800653 | 119.666871 | 29.068345 | 04/23/2021 17:01:08 | South Bus hub terminus |
| 1742800653 | 119.638016 | 29.128459 | 04/23/2021 17:54:21 | ZJNU |
| 1742800653 | 119.638016 | 29.128459 | 04/24/2021 10:03:56 | ZJNU |
| 1742800653 | 119.653573 | 29.116807 | 04/24/2021 10:15:07 | Jinhua Public Security Bureau |
| 1742800653 | 119.653573 | 29.116807 | 04/24/2021 13:03:58 | Jinhua Public Security Bureau |
| 1742800653 | 119.638016 | 29.128459 | 04/24/2021 13:20:51 | ZJNU |
| 1742800653 | 119.638016 | 29.128459 | 04/25/2021 06:30:56 | ZJNU |
| 1742800653 | 119.636916 | 29.112141 | 04/25/2021 06:55:07 | Jinhua High-speed Railway station |
| 1742800653 | 119.636916 | 29.112141 | 04/25/2021 07:59:09 | Jinhua High-speed Railway station |
| 1742800653 | 119.638016 | 29.128459 | 04/25/2021 08:20:51 | ZJNU |
| 1742800653 | 119.638016 | 29.128459 | 04/26/2021 07:00:06 | ZJNU |
| 1742800653 | 119.666871 | 29.068345 | 04/26/2021 07:57:07 | South Bus hub terminus |
| 1742800653 | 119.666871 | 29.068345 | 04/26/2021 11:03:58 | South Bus hub terminus |
| 1742800653 | 119.663557 | 29.076293 | 04/26/2021 11:15:40 | Traffic Police First squadron |
| 1742800653 | 119.663557 | 29.076293 | 04/26/2021 16:50:04 | Traffic Police First squadron |
| 1742800653 | 119.638016 | 29.128459 | 04/26/2021 17:56:51 | ZJNU |
| 1742800653 | 119.638016 | 29.128459 | 04/27/2021 07:10:56 | ZJNU |
| 1742800653 | 119.666871 | 29.068345 | 04/27/2021 08:07:07 | South Bus hub terminus |
| 1742800653 | 119.666871 | 29.068345 | 04/27/2021 16:03:58 | South Bus hub terminus |
| 1742800653 | 119.653573 | 29.116807 | 04/27/2021 16:55:40 | Jinhua Public Security Bureau |
| 1742800653 | 119.653573 | 29.116807 | 04/27/2021 16:13:04 | Jinhua Public Security Bureau |
| 1742800653 | 119.638016 | 29.128459 | 04/27/2021 16:32:51 | ZJNU |
| 1742800653 | 119.638016 | 29.128459 | 04/28/2021 06:23:43 | ZJNU |
| 1742800653 | 119.666871 | 29.068345 | 04/28/2021 07:15:24 | South Bus hub terminus |
| 1742800653 | 119.666871 | 29.068345 | 04/28/2021 16:53:58 | South Bus hub terminus |
| 1742800653 | 119.638016 | 29.128459 | 04/28/2021 17:43:51 | ZJNU |
| 1742800653 | 119.638016 | 29.128459 | 04/29/2021 07:00:56 | ZJNU |
| 1742800653 | 119.666871 | 29.068345 | 04/29/2021 07:51:45 | South Bus hub terminus |
| 1742800653 | 119.666871 | 29.068345 | 04/29/2021 17:03:12 | South Bus hub terminus |
| 1742800653 | 119.638016 | 29.128459 | 04/29/2021 18:20:43 | ZJNU |
| 1742800653 | 119.638016 | 29.128459 | 04/30/2021 06:53:21 | ZJNU |
| 1742800653 | 119.666871 | 29.068345 | 04/30/2021 07:45:54 | South Bus hub terminus |
| 1742800653 | 119.666871 | 29.068345 | 04/30/2021 15:23:58 | South Bus hub terminus |
| 1742800653 | 119.663557 | 29.076293 | 04/30/2021 15:55:11 | Traffic Police First squadron |
| 1742800653 | 119.663557 | 29.076293 | 04/30/2021 16:41:31 | Traffic Police First squadron |
| 1742800653 | 119.638016 | 29.128459 | 04/30/2021 17:53:51 | ZJNU |
