# Supplementary material for: Research on the method of travel area clustering of urban public transport based on Sage-Husa adaptive filter and improved DBSCAN algorithm
Source: PLoS One. 2021 Dec 22;16(12):e0259472. doi: 10.1371/journal.pone.0259472 (PMC8694428; doi:10.1371/journal.pone.0259472)
Supplement: S2 File — (DOCX) [file pone.0259472.s002.docx]

Supporting information

**S2. The layout of Jinhua city transit network (2020)**

**Fig 1. Transit network of Jinhua city (2020)**

| 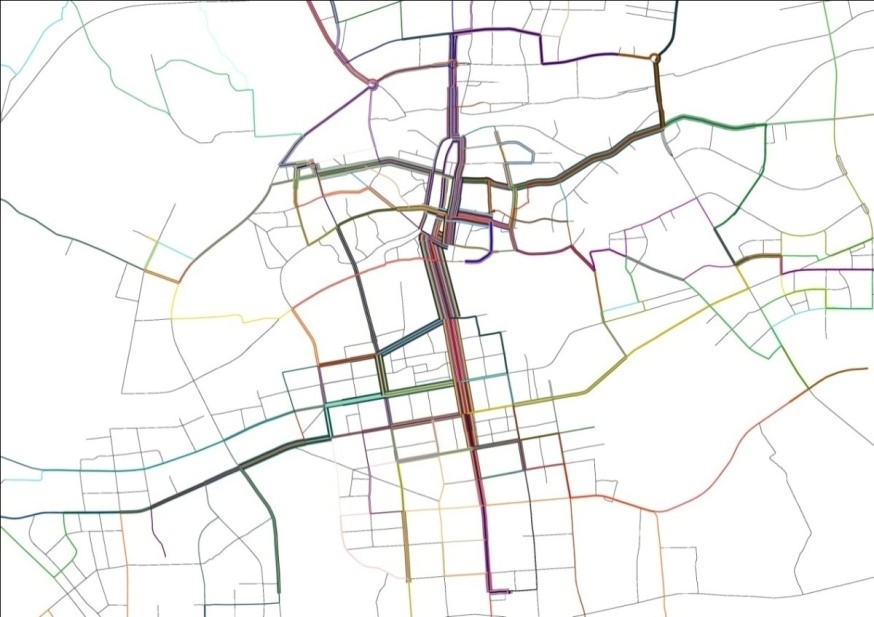 |
| --- |
| Fig 1. Transit network of Jinhua city |

**Fig 2.** **Cobweb of bus passenger flow in the central area (2020)**

| 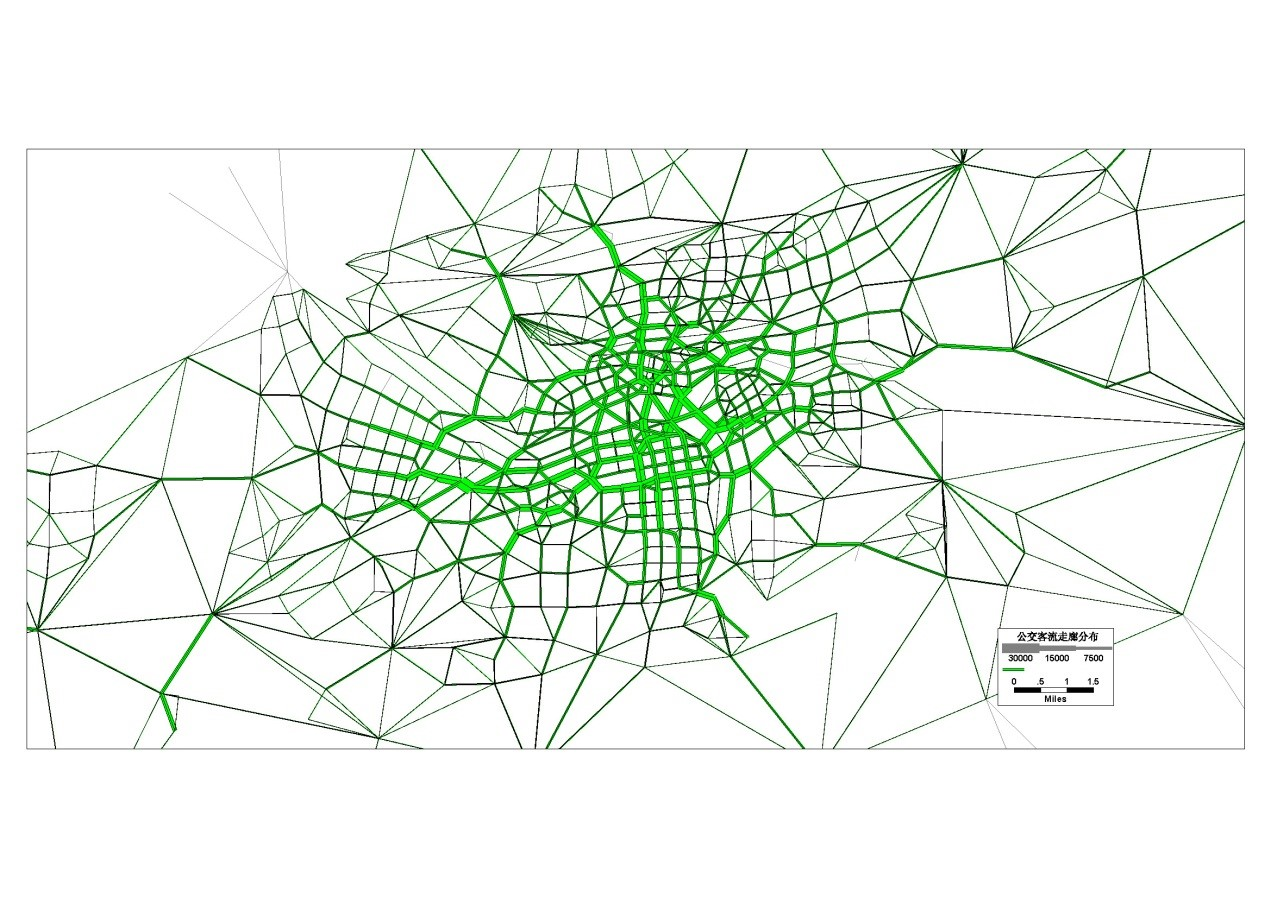 |
| --- |
| Fig 2. Cobweb of Bus Passenger Flow in the Central Area (2020) |

**Fig 3.** **Bus passenger flow in the central area (2020)**

| 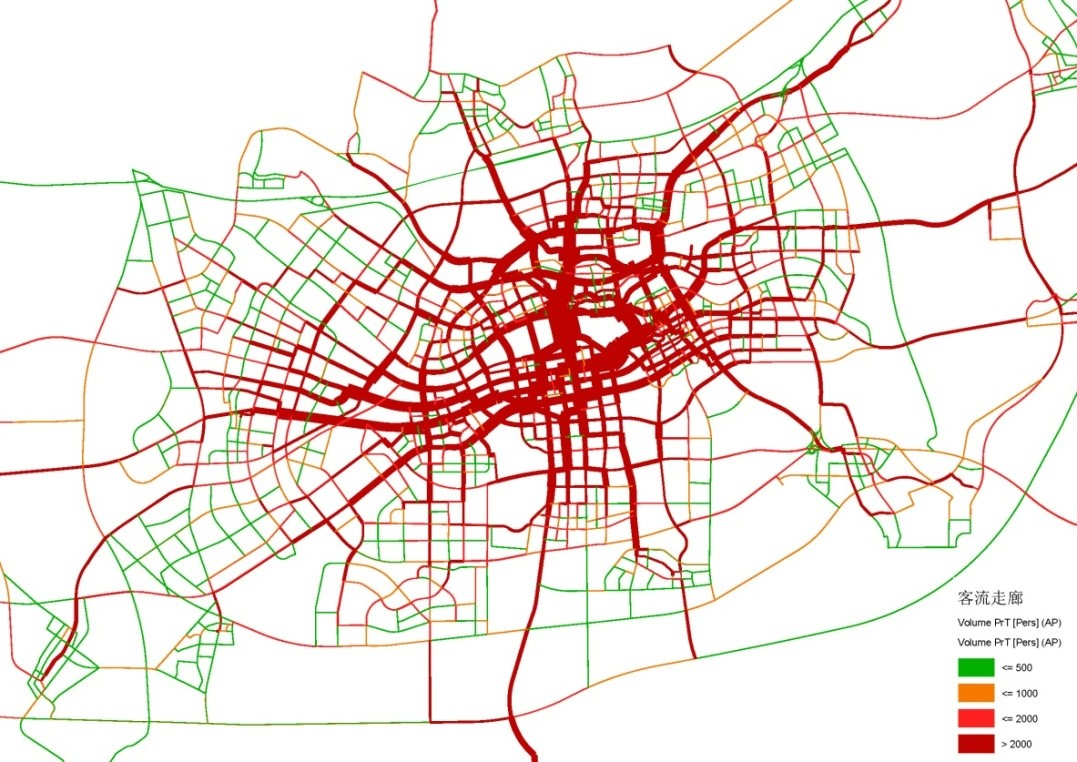 |
| --- |
| Fig 3. Bus Passenger Flow in the Central Area (2020) |

**Fig 4. Coverage of 300m bus stops in Jinhua city**

| 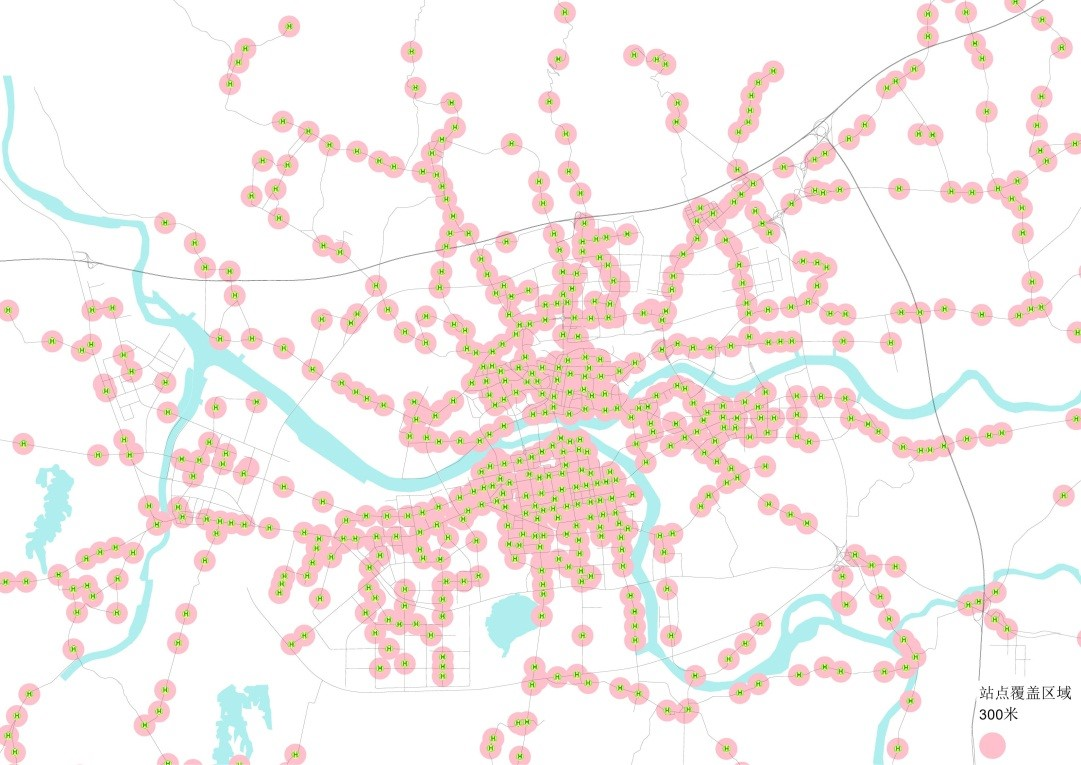 |
| --- |
| Fig 4. Coverage of 300m bus stops in Jinhua city |

**Fig 5. Coverage of 500m bus stops in Jinhua city**

| 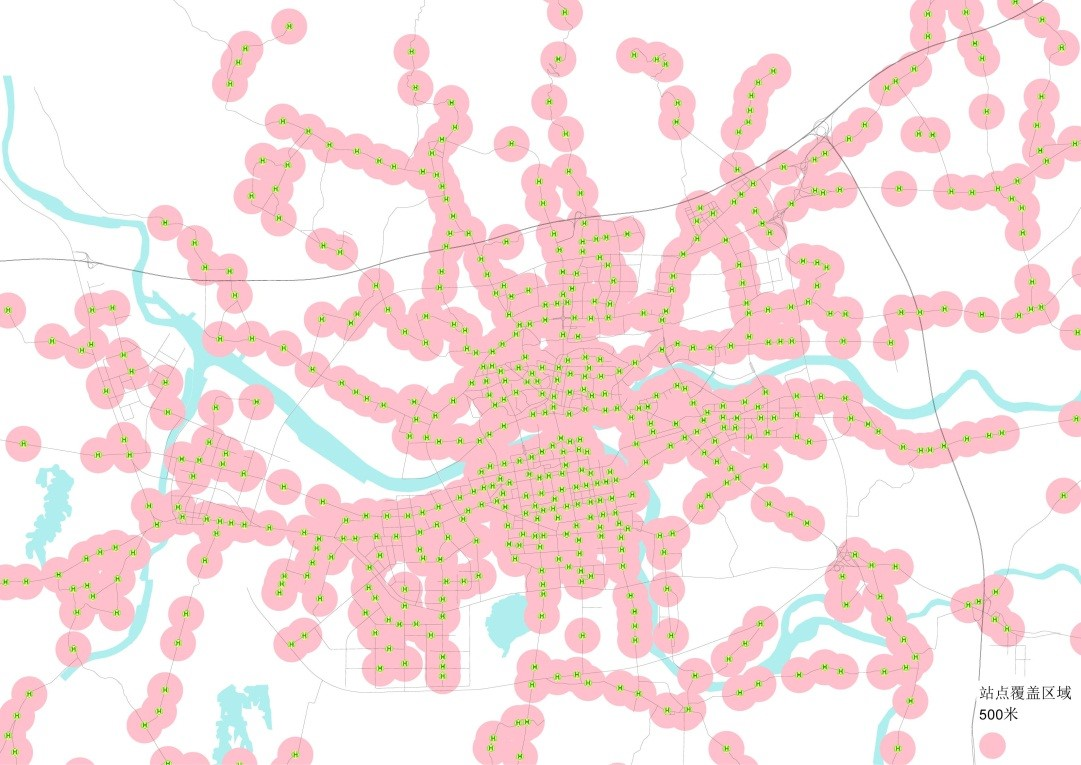 |
| --- |
| Fig 5. Coverage of 500m bus stops in Jinhua city |

**Table 1.** **transit index of Jinhua city (2020)**

| **Index** | **Unit** | **Status** | **Recommended Value** |
| --- | --- | --- | --- |
| Ten thousand people own vehicles | Standard vehicle | 7.65 | 10～12 |
| Average routes length | km | urban areas 16.6 | 8～12 |
|  |  | rural areas 21.0 |  |
| Density of network | km/km^2^ | the core area of ring one 3.01 | core area 3～4 |
|  |  | the core area of ring two 1.67 | peripheral area 2～2.5 |
| repetition coefficient of routes | -- | 3.27 | 1.25～2.5 |
| non-linear coefficient of routes | -- | urban areas 1.86 | <1.4 |
|  |  | rural areas 1.45 |  |
| Coverage of 300m | % | 49.6 | >50 |
| Coverage of 300m | % | 76.1 | >90 |
| Terminal facilities | m^2^/ Standard vehicle | 159 | -- |
| Average daily passenger capacity per vehicle | person-times / Standard vehicle | urban areas 339 | -- |
|  |  | rural areas 254 |  |
| Share rate of Public Transportation  (Including walking) | % | 8 | 20 |
| Average departure interval | min | 15～20 | -- |
| Average operating speed | Km/hour | Peak time 19.7 | >20 |
|  |  | Flat time 22.0 |  |
| Mean transfer coefficient | -- | 1.15 | Big city1.3-1.5 |
| Average station distance | Km / Boarded Times | 7.1 |  |
| Average travel time | Minutes/ Boarded Times | 26 |  |
| Average waiting time | min | 9 | 5 |

**Table 2.** **Station coverage of Jinhua center area**

| The service radius | Within ring one | Within ring two | The national standard |
| --- | --- | --- | --- |
| 300m | 74.9％ | 49.6％ | ≥50％ |
| 500m | 95.6％ | 76.1％ | ≥90％ |

**Table 3. Main comprehensive indexes of the transit network**

| **Routes** | **Departure interval (Min)** | **One-way (km)** | **Non-linear coefficient** | **Peak-time speed (km/h)** | **Total passenger flow** | **Full day load ratio** | **Daily passenger capacity**  **(Standard vehicle)** | **Revenue per kilometer**  **(RMB)** | **Average distance**  **(km)** |
| --- | --- | --- | --- | --- | --- | --- | --- | --- | --- |
| K9 | 10—15 | 16.9 | 1.47 | 14.9 | 5969 | 71.9% | 459 | 3.72 | 5.84 |
| K13 | 20--50 | 20.3 | 2.31 | 19.6 | 823 | 45.7% | 206 | 1.63 | 11 |
| K20 | 6—12 | 15.3 | 3.06 | 13.7 | 6839 | 44.3% | 489 | 3.43 | 5.84 |
| K26 | 10--20 | 10 | 1.61 | 17.6 | 1660 | 26.6% | 277 | 2.22 | 4.68 |
| K27 | 10—15 | 14.2 | 2.18 | 12.5 | 3674 | 63.6% | 408 | 3.76 | 5.76 |
| 32 | fixed | 8.1 | 1.5 |  | 159 | 33.5% | 114 | 1.67 | 3.92 |
| K35 | 8--20 | 10.6 | 1.63 | 14.5 | 2610 | 39.5% | 326 | 2.79 | 5.59 |
| K53 | 10--40 | 14.2 | 2.15 | 13.1 | 704 | 35.6% | 176 | 1.97 | 6.18 |
| 56 | 10--30 | 14.3 | 2.38 | 16.2 | 570 | 43.8% | 163 | 1.36 | 6.86 |
| K301 | 8—15 | 24.5 | 1.75 | 20.4 | 3969 | 27.7% | 235 | 2.12 | 8.3 |
| K307 | 20—70 | 29 | 1.29 |  | 501 | 30.1% | 114 | 1.13 | 13.33 |
| 309 | fixed | 13.8 | 1.55 |  | 368 | 31.9% | 184 | 1.85 | 7.32 |
| 310 | 20--60 | 28.6 | 2.86 | 24.5 | 1170 | 36.8% | 167 | 1.69 | 14.11 |
| 311 | fixed | 8 | 1.16 |  | 91 | 21.7% | 130 | 0.97 | 2.33 |
| 313 | fixed | 8.7 | 2.56 |  | 32 | 4.8% | 46 | 0.19 | 2.91 |
| 323 | fixed | 12 | 2.07 |  | 63 | 22.9% | 90 | 0.63 | 6.63 |
| 325 | 20-60 | 16.6 | 1.55 |  | 174 | 29.0% | 124 | 0.66 | 5.15 |
| Y6 | 10—25 | 13.6 | 1.68 | 13.6 | 3891 | 58.3% | 389 | 2.87 | 5.71 |
| Y7 | 15--40 | 25.7 | 1.41 | 20.6 | 2778 | 44.1% | 237 | 3.11 | 11.24 |
| K1 | 10--30 | 12.2 | 2.49 | 24.4 | 80 | 9.2% | 38 | 0.39 | 5.21 |
| K8 | 10--15 | 12.9 | 1.65 | 15.5 | 3771 | 38.0% | 322 | 3.93 | 5.54 |
| K11 | 10--15 | 13.5 | 1.99 | 18.8 | 4737 | 34.8% | 331 | 3.72 | 5.96 |
| K12 | 7--15 | 10.5 | 1.54 | 15.8 | 5789 | 36.9% | 445 | 4.89 | 5.16 |
| K28 | 10--15 | 13.1 | 3.05 | 17.5 | 2425 | 29.4% | 266 | 2.79 | 5.94 |
| K36 | 10--15 | 12.5 | 2.98 | 16.3 | 4701 | 31.6% | 329 | 3.46 | 5.3 |
| 54 | fixed | 10.8 | 1.4 | 13 | 140 | 38.9% | 200 | 1.37 | 5.66 |
| 127 | 30--45 | 10.6 | 1.74 | 12.7 | 161 | 16.3% | 81 | 1.15 | 4.28 |
| 303 | 40-60 | 19.8 | 1.87 | 23.8 | 502 | 46.5% | 251 | 1.96 | 7.89 |
| 308 | fixed | 26.8 | 2.79 | 35.7 | 90 | 8.3% | 45 | 0.28 | 10.08 |
| 315 | 12--20 | 13.6 | 1.23 | 17 | 3069 | 40.6% | 307 | 2.16 | 5.64 |
| 318 | fixed | 24.5 | 1.99 | 24.5 | 237 | 107.7% | 119 | 4.92 | 14.56 |
| 352 | 20-45 | 12.3 | 1.41 | 18.5 | 645 | 29.9% | 129 | 1.82 | 4.18 |
| Y3 | 40--60 | 27.9 | 1.69 | 27.9 | 403 | 15.7% | 78 | 0.82 | 9.99 |
| Y5 | fixed | 25.5 | 2.14 | 30.6 | 218 | 15.1% | 84 | 0.78 | 12.98 |
| Y 9 | 20-30 | 21.8 | 1.96 | 26.2 | 1157 | 30.9% | 145 | 1.38 | 9.66 |
| K15 | 8—15 | 11.9 | 1.83 | 14.3 | 3925 | 36.6% | 393 | 3.07 | 4.35 |
| k16 | fixed | 17 | 1.72 | 20.4 | 82 | 32.8% | 117 | 0.88 | 6.17 |
| K18 | 15--20 | 10.9 | 2.37 | 16.4 | 3403 | 60.1% | 681 | 4.22 | 4.5 |
| K19 | 8--15 | 8.7 | 1.5 | 14.9 | 4827 | 53.8% | 536 | 4.93 | 4.95 |
| K24 | 8—15 | 14.2 | 1.89 | 14.7 | 6900 | 49.0% | 408 | 4.37 | 5.37 |
| 33 | 12--30 | 12.6 | 1.48 | 15.8 | 842 | 30.2% | 168 | 1.87 | 5.22 |
| 34 | 20--60 | 14.7 | 1.28 | 17.6 | 279 | 23.8% | 101 | 1.3 | 5.13 |
| K37 | 10--15 | 14.3 | 2.1 | 15.6 | 5128 | 35.1% | 394 | 3.79 | 6.45 |
| K38 | 10--20 | 12.2 | 2.07 | 17 | 2386 | 41.8% | 341 | 2.56 | 4.55 |
| 302 | 30--60 | 17.6 | 1.47 | 26.4 | 616 | 55.8% | 205 | 2.57 | 6.5 |
| 304 | 30--60 | 19.3 | 1.72 | 23.2 | 413 | 11.3% | 59 | 0.48 | 7.42 |
| 306 | 20--60 | 14 | 1.67 | 16.8 | 500 | 24.2% | 167 | 1.54 | 7.36 |
| 314 | 20--30 | 23.5 | 2.24 | 18.8 | 1706 | 71.3% | 284 | 2.3 | 11.55 |
| 317 | 20--60 | 14.9 | 1.52 | 22.4 | 492 | 30.8% | 164 | 1.61 | 6.3 |
| 322 | 20--50 | 35 | 2.27 | 35 | 1487 | 78.7% | 313 | 1.92 | 15.41 |
| 326 | 20--50 | 45.1 | 3.01 | 45.1 | 2169 | 51.9% | 241 | 1.19 | 18.49 |
| K330 | 9--15 | 13.8 | 1.62 | 15.1 | 6548 | 60.1% | 546 | 3.77 | 5.56 |
| 339 | fixed | 17.1 | 1.78 | 17.1 | 236 | 35.0% | 236 | 0.94 | 7.02 |
| 350 | fixed | 10.5 | 1.54 | 15.8 | 151 | 37.3% | 216 | 1.72 | 3.9 |
| 521 | fixed | 30 | 1.13 |  | 441 | 26.5% | 110 | 1.44 | 11.82 |
| 522 | 15—40 | 30.2 | 1.2 |  | 2262 | 55.9% | 283 | 2.88 | 12.98 |
| 523 | 15—40 | 28.6 | 1.41 |  | 1805 |  | 1805 | 17.16 | 9.57 |
| 524 | 20—40 | 21.6 | 1.77 |  | 628 | 30.3% | 157 | 1.4 | 9.38 |
| 527 | 15-40 | 16.4 | 1.2 |  | 1633 | 54.2% | 233 | 2.59 | 5.98 |
| 528 | 15-40 | 27.5 | 1.14 |  | 3409 | 64.2% | 213 | 2.68 | 9.09 |
| 631 | 30-60 | 9.1 | 1.28 |  | 407 |  | 407 | 22.12 | 10.38 |
| 632 | 20-60 | 11.7 | 2.66 |  | 959 | 44.4% | 240 | 1.7 | 8.21 |
| 633 | 30-60 | 13.6 | 1.4 |  | 335 | 49.6% | 168 | 2.11 | 6.19 |
| 635 | fixed | 14.7 | 1.25 |  | 315 | 21.9% | 105 | 0.84 | 7.99 |
| 642 | fixed | 4.6 | 1.12 |  | 100 | 13.9% | 100 | 1.36 |  |
| 643 | 20—40 | 29.7 | 1.34 |  | 984 | 37.7% | 141 | 1.66 | 8.36 |
| 510 | 10—30 | 23.5 | 1.37 |  | 1716 | 38.5% | 172 | 1.67 | 8.95 |
| 505 | 8-15 | 27 | 1.62 |  | 2882 | 49.3% | 192 | 2.3 | 11.82 |
| 508 | 10—20 | 13.9 | 1.9 |  | 2103 | 33.1% | 234 | 2.12 | 5.19 |
| 525 | 20_40 | 23 | 1.46 |  | 1128 | 35.8% | 141 | 1.54 | 10.92 |
| 529 | 20—50 | 22.3 | 1.45 |  | 500 | 33.7% | 125 | 1.68 | 8.71 |
| 530 | 10—30 | 15.9 | 1.92 |  | 997 | 33.6% | 166 | 1.9 | 6.84 |
| 601 | fixed | 50.7 | 1.86 |  | 153 | 42.5% | 77 | 1.31 | 20.4 |
| 602 | fixed | 56 | 1.69 |  | 51 | 28.3% | 51 | 0.91 | 2.07 |
| 630 | fixed | 9.2 | 1.33 |  | 75 | 11.9% | 75 | 0.71 | 3.74 |
| 501 | 30-40 | 33.3 | 1.31 |  | 739 | 31.0% | 123 | 1.39 | 14.4 |
| 502 | 7-15 | 31.2 | 1.17 |  | 4575 | 51.9% | 183 | 2.79 | 13.4 |
| 503 | 7-20 | 40.5 | 1.28 |  | 3236 | 69.8% | 180 | 2.93 | 13.79 |
| 506 | 15-55 | 9.4 | 1.27 |  | 269 | 24.9% | 135 | 2.33 | 0.67 |
| 507 | 60-100 | 10.2 | 1.34 |  | 78 | 10.8% | 78 | 0.95 | 6.57 |
| 604 | 20-60 | 18 | 1.44 |  | 251 | 29.4% | 126 | 0.89 | 4.2 |
| 611 | 15-40 | 17 | 1.35 |  | 91 | 18.4% | 91 | 1.11 | 3.39 |
| 612 | 15-40 | 20 | 1.6 |  | 106 | 19.6% | 106 | 0.85 | 1.05 |
| 613 | 15-40 | 13 | 1.31 |  | 303 | 15.3% | 101 | 0.95 | 1 |
| 621 | 10-70 | 13.4 | 1.33 |  | 156 | 14.4% | 78 | 0.71 | 7.21 |
| 603 | 20-50 | 8.1 | 1.21 |  | 167 | 12.0% | 84 | 0.8 | 3.37 |
| 605 | fixed | 54.5 | 1.85 |  | 71 | 52.6% | 71 | 1.48 |  |
| 6009 | 20-40 | 27 | 1.09 |  | 545 | 25.2% | 78 | 1.16 | 4.52 |
